# Synergistic activation of mutant *TERT* promoter by Sp1 and GABPA in BRAF^V600E^-driven human cancers

**DOI:** 10.1038/s41698-020-00140-5

**Published:** 2021-01-22

**Authors:** Yongxing Wu, Liang Shi, Yuelei Zhao, Pu Chen, Rongrong Cui, Meiju Ji, Nongyue He, Maode Wang, Gang Li, Peng Hou

**Affiliations:** 1 Key Laboratory for Tumor Precision Medicine of Shaanxi Province and Department of Endocrinology, The First Affiliated Hospital of Xi’an Jiaotong University, 710061 Xi’an, People’s Republic of China; 2 Department of Critical Care Medicine, The First Affiliated Hospital of Xi’an Jiaotong University, 710061 Xi’an, People’s Republic of China; 3 Center for Translational Medicine, The First Affiliated Hospital of Xi’an Jiaotong University, 710061 Xi’an, People’s Republic of China; 4 State Key Laboratory of Bioelectronics, Southeast University, 210096 Nanjing, People’s Republic of China; 5 Department of Neurosurgery, The First Affiliated Hospital of Xi’an Jiaotong University, 710061 Xi’an, People’s Republic of China; 6 Department of Neurosurgery, Tangdu Hospital, Fourth Military Medical University, 710038 Xi’an, People’s Republic of China

**Keywords:** Cancer genetics, Oncogenes

## Abstract

The activating *TERT* promoter mutations and *BRAF*^*V600E*^ mutation are well-established oncogenic alterations in human cancers. Coexistence of *BRAF*^*V600E*^ and *TERT* promoter mutations is frequently found in multiple cancer types, and is strongly associated with poor patient prognosis. Although the BRAF^V600E^-elicited activation of ERK has been demonstrated to contribute to TERT reactivation by maintaining an active chromatin state, it still remains to be addressed how activated ERK is selectively recruited to mutant *TERT* promoter. Here, we report that transcription factor GABPA mediates the regulation of BRAF^V600E^/MAPK signaling on TERT reactivation by selectively recruiting activated ERK to mutant *TERT* promoter, where activated ERK can phosphorylate Sp1, thereby resulting in HDAC1 dissociation and an active chromatin state. Meanwhile, phosphorylated Sp1 further enhances the binding of GABPA to mutant *TERT* promoter. Taken together, our data indicate that GABPA and Sp1 synergistically activate mutant *TERT* promoter, contributing to tumorigenesis and cancer progression, particularly in the BRAF^V600E^-driven human cancers. Thus, our findings identify a direct mechanism that bridges two frequent oncogenic alterations together in TERT reactivation.

## Introduction

Telomeres are special DNA-protein structures located at both ends of eukaryotic chromosomes, protecting genomic material and sheltering chromosome ends from DNA damage response machinery^1^. Since telomeres shorten with every cell division, telomerase re-expression is required for maintaining stable telomeres and permissive for the indefinite cell growth in most advanced malignancies^2,3^. Telomerase reverse transcriptase (TERT) encodes the catalytic subunit of telomerase, and TERT reactivation is a key step in telomerase re-expression. Recently, there are many studies showing that TERT potentiates the oncogenesis not only by catalyzing the elongation of telomeres, which is the primary function of telomerase, but also by conferring proliferation advantages through directly regulating MYC stability and tRNA transcription^4,5^, suggesting its critical role in tumorigenesis and tumor progression.

Since TERT reactivation has played a pivotal role in maintaining the telomeres and thereby supporting infinite cell division in cancer cells, many researchers have sought to determine the genetic basis for TERT reactivation in different types of cancer. It should be noted that *TERT* promoter mutations are frequently found in multiple cancer types and predict poor patient prognosis^6,7^. In particular, two hotspot somatic mutations precisely located at positions −124 or −146 bp upstream of the TERT transcription start site (−124 C > T and −146 C > T, also known as C228T and C250T) were documented each to create a de novo ETS binding site (EBS), and enhanced transcriptional activity of *TERT* promoter^7^. Further studies demonstrated that these hotspot mutations selectively recruited transcription factor GABPA to promote *TERT* transcription^8^. In addition, mutant *TERT* promoter exhibited active chromatin marks, while its wild-type allele remained an inactive chromatin state^9^, indicating that these mutations initiated an epigenetic switch and the mono-allelic expression of TERT. There is also a study reporting that binding of GABPA to mutant *TERT* promoter can mediate the long-range chromatin interactions, facilitating the acquisition of an active chromatin state of *TERT* promoter and enhancing *TERT* transcription^10^. Collectively, these studies have highlighted the importance of *TERT* promoter mutations as a gate-keeper for TERT reactivation and tumor progression.

*BRAF*^*V600E*^ mutation is another frequent genetic alteration that drives tumorigenesis and tumor progression, particularly in thyroid cancers and melanomas^11,12^. This mutation results in constitutively activating BRAF kinase, contributing to phosphorylation of the downstream mitogen-activated protein kinases (MAPK)^13^. The abnormal activation of BRAF signals regulates various pathways and has a vital role in tumorigenesis, and recent studies demonstrated that inhibitors targeting BRAF pathway showed promising clinical activities^14,15^. Co-occurrence of *BRAF*^*V600E*^ and *TERT* promoter mutations has been found in 7.1–9.8% of papillary thyroid cancers (PTCs) and 13.6–20.7% melanomas^16,17^, which is closely correlated with worse prognosis and more aggressive pathological characteristics, suggesting a cooperative role between BRAF^V600E^ signaling and *TERT* promoter mutations in cancer initiation and progression. A recent study has demonstrated that GABPB1L, a catalytic subunit of GABP, positively regulates TERT expression in a *TERT* promoter mutation-dependent manner^18^. Moreover, there is evidence indicating that the BRAF^V600E^/MAPK cascade enhances GABPB transcription by phosphorylating transcription factor Fos, thereby activating mutant *TERT* promoter through forming a hetero-tetrameric complex with GAPBA^19^. Another study has also revealed that the constitutively activated BRAF^V600E^/ERK signal can selectively regulate an active chromatin state on mutant *TERT* promoter by phosphorylating Sp1 and facilitating the dissociation of HDAC1^20^. These observations indicate a direct link between *BRAF*^*V600E*^ and *TERT* promoter mutations in activating *TERT* transcription. However, questions have been raised on how activated ERK selectively binds to mutant *TERT* promoter, while not its wild-type allele.

In this study, we attempt to fill this gap by testing the hypothesis that activated ERK may be recruited to mutant *TERT* promoter by pioneer transcription factors. We demonstrate that transcription factor GABPA is required for recruiting activated ERK to mutant *TERT* promoter, thereby resulting in Sp1 phosphorylation, the consequent dissociation of HDAC1 and TERT activation. Our data also show that activated ERK signaling can enhance the binding of GABPA to mutant *TERT* promoter in an Sp1-dependent manner. Altogether, our results reveal that GABPA bridges the *BRAF*^*V600E*^ and *TERT* promoter mutations together in TERT reactivation, and demonstrate that GABPA and Sp1 synergistically transactivate mutant *TERT* promoter.

## Results

### Activated ERK is recruited to mutant *TERT* promoter by the GABP tetramer

**Fig. 1:**
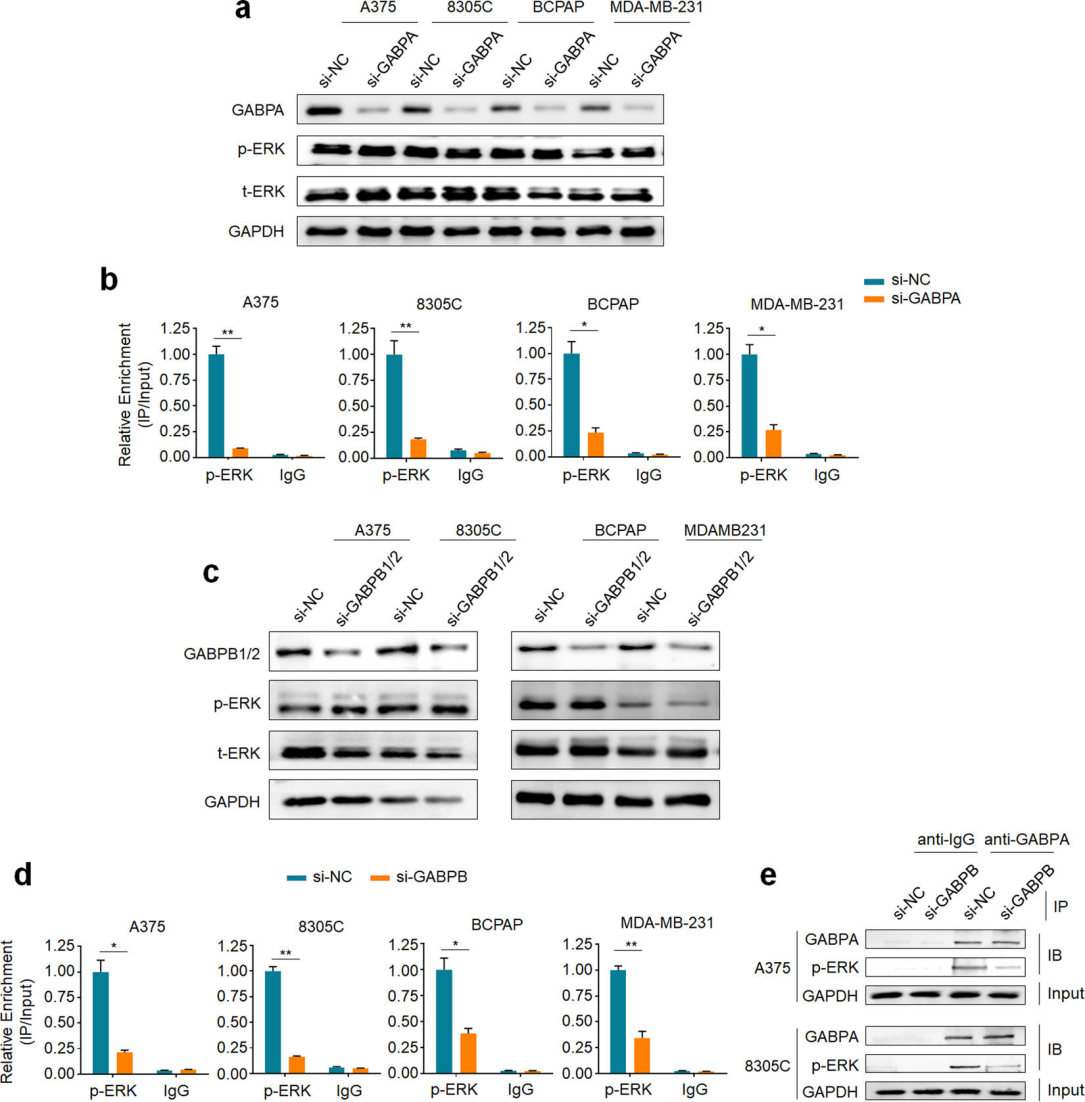
GABP tetramer mediates the recruitment of activated ERK to mutant *TERT* promoter. **a** Western blot was performed to determine the effect of GABPA knockdown on the levels of phosphorylated ERK (p-ERK) and total ERK (t-ERK) in the indicated cell lines. **b** ChIP-qPCR assay was performed to evaluate the effect of knocking down GABPA in the indicated cancer cells on the binding of p-ERK to *TERT* promoter. **c** Western blot was used to assess the effect of GABPB1/2 knockdown on the levels of p-ERK and t-ERK in the indicated cell lines. **d** ChIP-qPCR assay was performed to determine the effect of GABPA knockdown on the binding of p-ERK to *TERT* promoter in the indicated cells. **e** Co-IP with indicated antibodies followed by immunoblot (IB) showing the interaction between GABPA and p-ERK in A375 and 8305C cells knocking down GABPB and control cells. Data were shown as mean ± SD. *P < 0.05; **P < 0.01.

Given that GABPA can selectively bind to de novo EBS created by C250T or C228T *TERT* promoter mutation^8^, and GABPA is a direct downstream target of MAPK/ERK signaling^21^. Besides, there is evidence showing that ERK can be recruited to specific target sequences and exert its function by its downstream target genes^22^. These observations motivated us to assume that GABPA may recruit activated ERK (phosphorylated ERK, p-ERK) to mutant *TERT* promoter, leading to the activation of *TERT* promoter. To prove this, we first determined the effect of GABPA depletion on protein expression and phosphorylation of ERK in four cancer cell lines harboring both *BRAF*^*V600E*^ and *TERT* promoter mutations, including melanoma cell line A375, thyroid cancer cell lines BCPAP and 8305C, and breast cancer cell line MDA-MB-231. As shown in Fig. 1a, GABPA knockdown in these cells almost did not affect ERK expression and phosphorylation, while caused a substantial decline in p-ERK recruitment at mutant *TERT* promoter (Fig. 1b), indicating that GABPA should be involved in regulating the recruitment of p-ERK to mutant *TERT* promoter.

GABP is composed of two distinct but functional related subunits. The alpha subunit of GABP (GABPA) only includes the DNA-binding domain (DBD), while the beta subunit of GABP (GABPB) contains the transcription activation domain (TAD)^23^. Heterodimerization or heterotetramerization of GABPA and GABPB is required for GABP to exert its full transcription activity. Thus, we next determined whether GABPB was also involved in p-ERK recruitment. As expected, knocking down GABPB in A375 and 8305C cells almost did not change expression and phosphorylation of ERK (Fig. 1c), while caused a significant decrease in p-ERK recruitment at mutant *TERT* promoter (Fig. 1d), indicating that both subunits of GABP are required for the recruitment of p-ERK to mutant *TERT* promoter. Next, we attempted to determine whether there exists an interaction between GABPA/GABPB complex and p-ERK. Using co-IP assay, we observed a strong interaction between GABPA and p-ERK, while knocking down GABPB in A375 and 8305C cells markedly attenuated their interaction (Fig. 1e), suggesting that p-ERK is more likely to interact with GABPB rather than GABPA. A recent study has demonstrated that the constitutively activated RAS/ERK signal selectively regulates an active chromatin state on mutant *TERT* promoter, while the conversion of *TERT* promoter mutations to wild-type promoter can abolish this effect, indicating that de novo ETS binding site is required for this novel regulatory interaction^20^. Taken together, our data indicate that the GABP tetramer is required for the regulation of mutant *TERT* promoter by BRAF^V600E^/MAPK/ERK signaling.

### ERK regulates the binding of GABPA to mutant *TERT* promoter via Sp1

**Fig. 2:**
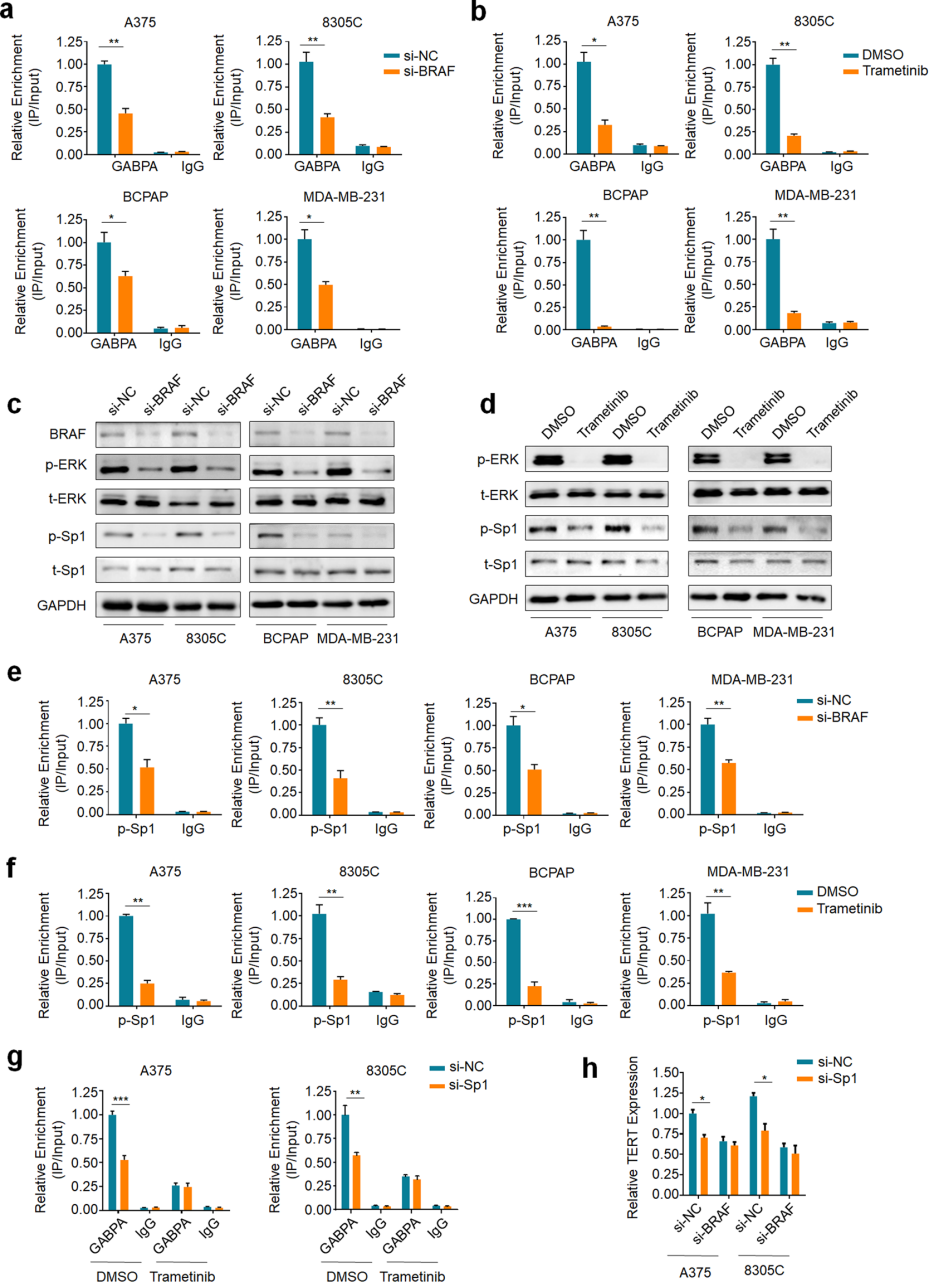
The BRAF^V600E^/ERK/Sp1 signaling enhances the recruitment of GABPA to mutant *TERT* promoter. ChIP-qPCR assay was performed in the indicated cells to evaluate the effect of inhibition of ERK activation by BRAF knockdown (**a**) or trametinib treatment (**b**) on the binding of GABPA to mutant *TERT* promoter. Western blot analysis was performed in the indicated cells to evaluate the effect of BRAF knockdown (**c**) or trametinib treatment (**d**) on phosphorylation of ERK and Sp1. GAPDH was used as a loading control. ChIP-qPCR assay was performed in the indicated cells to test the effect of BRAF knockdown (**e**) or trametinib treatment (**f**) on the binding of p-Sp1 to mutant *TERT* promoter. **g** ChIP-qPCR assay was performed to determine the effect of Sp1 knockdown on the recruitment of GABPA to mutant *TERT* promoter in the indicated cells treated with trametinib or si-HDAC1. **h** qRT-PCR assay was performed to determine the effect of Sp1 knockdown on *TERT* expression in the indicated cells knocking down BRAF or control cells. *18S* rRNA was used as a reference gene. Data were shown as mean ± SD. *P < 0.05; **P < 0.01; ***P < 0.001.

Next, we attempted to determine whether BRAF^V600E^-mediated MAPK/ERK signaling can affect the binding capacity of GABP to mutant *TERT* promoter. To address this, we treated A375, 8305C, BCPAP, and MDA-MB-231 cells with siRNA targeting BRAF (si-BRAF) or 100 nM MEK inhibitor trametinib for 6 or 24 h to block the ERK cascade. The results showed that treatment with si-BRAF or trametinib markedly inhibited ERK phosphorylation, while almost did not change GABPA expression (Supplementary Fig. 1a, b). However, the ChIP assay showed that inhibition of the BRAF^V600E^/ERK signaling by si-BRAF (Fig. 2a) or trametinib (Fig. 2b) could significantly reduce the binding of GABPA to mutant *TERT* promoter in the indicated cells.

There is evidence showing that both subunits of GABP can be phosphorylated by MAPK/ERK signaling; however, in vitro assay showed that DNA-binding capacity of GABP heterodimer or heterotetramer was almost not affected by ERK-elicited phosphorylation^24^. Considering that the presence of GABPB and the consequent formation of GABPA/B heterodimer may stabilize the GABPA–DNA interaction^25^, and the MAPK/ERK signaling upregulated GABPB expression via Fos phosphorylation^19^, thus we first validated the effect of ERK cascade on GABPB expression. Expectedly, using qRT-PCR assay, we found that treatment of A375, 8305C, BCPAP, and MDA-MB-231 cells with 100 nM trametinib could inhibit the expression of GABPB1, but not GABPB2, particularly for a 24-h treatment (Supplementary Fig. 1c). This was also supported by western blot results (Supplementary Fig. 1d), which was consistent with a previous study^19^.

We attempted to explore the mechanism of ERK activation affecting the binding of GABPA to *TERT* promoter. There are many studies demonstrating that the binding capacity and specificity of ETS family members to the targeting sequence can be regulated by protein–protein interactions with co-regulatory partners, such as CBP/p300, Sp1 and PU.1^26–30^. Coincidently, we found close proximity of the binding sites of GABP and Sp1 on mutant *TERT* promoter. Thus, it will prompt us to test the possibility that transcription factor Sp1, a downstream target of the MAPK/ERK signaling, may serve as an enhancer for GABPA binding. First, we determined whether ERK signaling could regulate the phosphorylation status of Sp1 at *TERT* promoter. Similar to a previous study^20^, inhibition of BRAF^V600E^/ERK signaling by si-BRAF or trametinib almost did not change the expression of total Sp1 (t-Sp1), while markedly decreased the levels of phosphorylated Sp1 (p-Sp1) (Fig. 2c, d). Correspondingly, the blockade of BRAF^V600E^/ERK signaling could reduce the recruitment of p-Sp1 to *TERT* promoter (Fig. 2e, f), indicating that p-Sp1 may be involved in ERK-induced GABPA binding in cancer cells. Besides, the specificity of ChIP-qPCR was validated by determining the binding capacity of GABPA/Sp1 to the region lacking a corresponding binding site (Supplementary Fig. 2).

Next, we aimed to investigate the role of Sp1 in regulating the binding capacity of GABPA. As shown in Supplementary Fig. 3, Sp1 knockdown did not affect GABPA expression and ERK phosphorylation, while significantly reduced the occupancy of GABPA on mutant *TERT* promoter in the indicated cells, and this effect could be abolished by trametinib treatment (Fig. 2g). Meanwhile, we attempted to determine the effect of Sp1 knockdown alone or in combination with HDAC knockdown on GABPA enrichment at mutant *TERT* promoter. As shown in Fig. 2g, knocking down HDAC1 remarkably increased the enrichment of GABPA at mutant *TERT* promoter compared to the control. However, dual knockdown of HDAC1 and Sp1 almost did not affect GABPA enrichment at mutant *TERT* promoter. These results further support our conclusion that HDAC1 is recruited to Sp1 in the ERK activated cancer cells, sterically hindering GABPA enrichment at mutant *TERT* promoter. In addition, we found that inhibition of Sp1 expression remarkably decreased TERT expression, while this effect was expectedly abolished by BRAF downregulation (Fig. 2h). To further determine whether the regulation of GABPA binding capacity by Sp1 depends on the *TERT* promoter mutations, we knocked down Sp1 in gastric cancer cell line AGS and colon cancer cell line RKO carrying *BRAF*^*V600E*^ mutation and wild-type *TERT* promoter. The results showed that Sp1 knockdown only minimally affected the binding of GABPA to *TERT* promoter (Supplementary Fig. 4), indicating that the regulation of GABPA binding capacity by Sp1 depends on *TERT* promoter mutations. Altogether, our results indicate that Sp1 may mediate p-ERK-elicited enhancement of GABPA to mutant *TERT* promoter.

### Sp1 phosphorylation-mediated HDAC1 dissociation leads to enhanced binding of GABPA to mutant *TERT* promoter

**Fig. 3:**
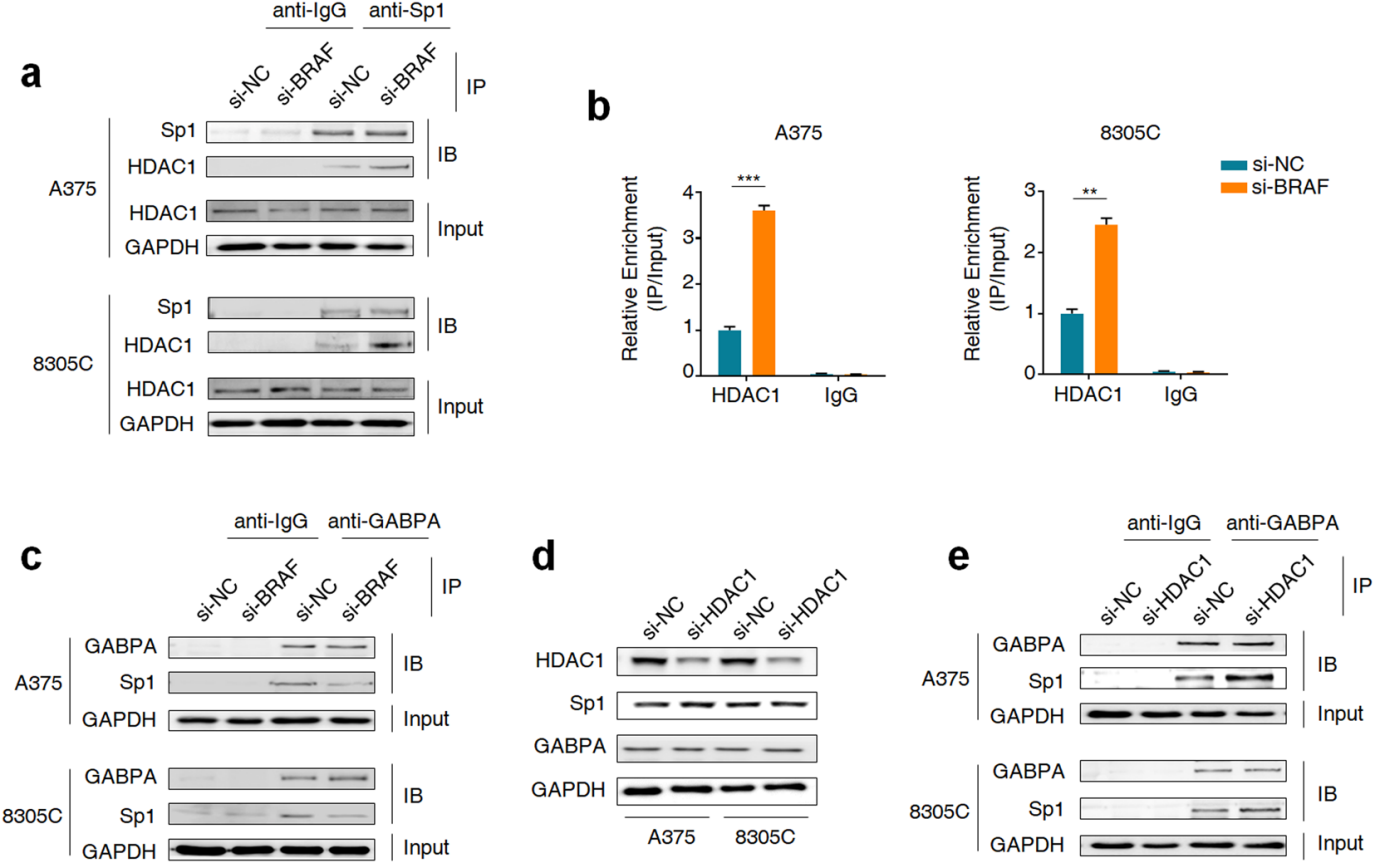
HDAC1 poses steric hindrance for the interaction between GABPA and Sp1. **a** Co-IP assay of whole-cell lysates derived from the indicated cells knocking down BRAF and control cells to validate the interaction between Sp1 and HDAC1. **b** ChIP-qPCR assay was performed to evaluate the effect of inhibition of BRAF^V600E^-mediated ERK activation on the binding of HDAC1 to *TERT* promoter in the indicated cells knocking down BRAF and control cells. **c** Co-IP assay was similarly performed in the indicated cells knocking down BRAF and control cells to validate the interaction between Sp1 and GABPA. **d** Western blot analysis was performed to determine the effect of HDAC1 knockdown on the expression and GABPA and Sp1 in the indicated cells. **e** Co-IP assay was performed to determine the effect of HDAC1 knockdown on the interaction between Sp1 and GABPA. Data were shown as mean ± SD. **P < 0.01; ***P < 0.001.

We attempted to reveal the mechanism of Sp1 regulating the recruitment of GABPA to mutant *TERT* promoter. There are studies demonstrating that HDAC1 and GABPA both interact with the zinc finger DNA-binding domain of Sp1^31,32^, and activated ERK binding to *TERT* promoter can lead to Sp1 phosphorylation and the consequent dissociation of HDAC1^20,22^. Thus, we speculate that BRAF^V600E^-mediated ERK signaling may promote the interaction between Sp1 and GABPA by relieving steric hindrance of HDAC1. To prove this, we performed co-IP assay to test the interaction between HDAC1 and Sp1 in A375 and 8305C cells knocking down BRAF and control cells. The results showed that, compared to the control, BRAF knockdown almost did not affect HDAC1 expression, while clearly enhanced the interaction between Sp1 and HDAC1 (Fig. 3a), thereby promoting the recruitment of HDAC1 to *TERT* promoter (Fig. 3b). Meanwhile, we also found that BRAF knockdown attenuated the interaction between Sp1 and GABPA (Fig. 3c). As supported, trametinib treatment similarly promoted the recruitment of HDAC1 to *TERT* promoter by enhancing the interaction between Sp1 and HDAC1 (Supplementary Fig. 5a, b), and attenuated the interaction between Sp1 and GABPA (Supplementary Fig. 5c) compared to the control.

To be consistent with the above findings, we knocked down HDAC1 in A375 and 8305C cells, and found that HDAC1 depletion almost did not change the expression of Sp1 and GABPA (Fig. 3d), while substantially enhanced the interaction between Sp1 and GABPA (Fig. 3e). In addition, we knocked down HDAC1 in RKO and AGS cells carrying *BRAF*^*V600E*^ mutation and wild-type *TERT* promoter. The results showed that HDAC1 knockdown did not affect GABPA expression and its recruitment at *TERT* promotor (Supplementary Fig. 6), indicating that regulatory effect of HDAC1 on GABPA binding capacity is dependent on *TERT* promoter mutation status. Collectively, these results demonstrate that BRAF^V600E^-mediated ERK signaling promotes the recruitment of GABPA to mutant *TERT* promoter via Sp1 phosphorylation-mediated HDAC1 dissociation.

### Sp1 phosphorylation is crucial for GABPA binding and TERT activation

Given that the ERK-elicited phosphorylation of Sp1 is crucial for GABPA binding at *TERT* promoter, we next sought to determine the role of the ERK-dependent phosphorylation sites of Sp1 in regulating the recruitment of GABPA to mutant *TERT* promoter. There is evidence revealing that activated ERK can directly phosphorylate Sp1 at serine residue(s) and threonines 453 and 739 (T453 and T739)^33^. T453 residue in the BQ region is a transactivating domain at the N-terminus of Sp1, while T739 residue is located in the D region, mediating the interaction between Sp1 and other transcription factors^34,35^. In addition, phosphorylation at T739 residue has been supposed to contribute to further Sp1 phosphorylation^36^.

**Fig. 4:**
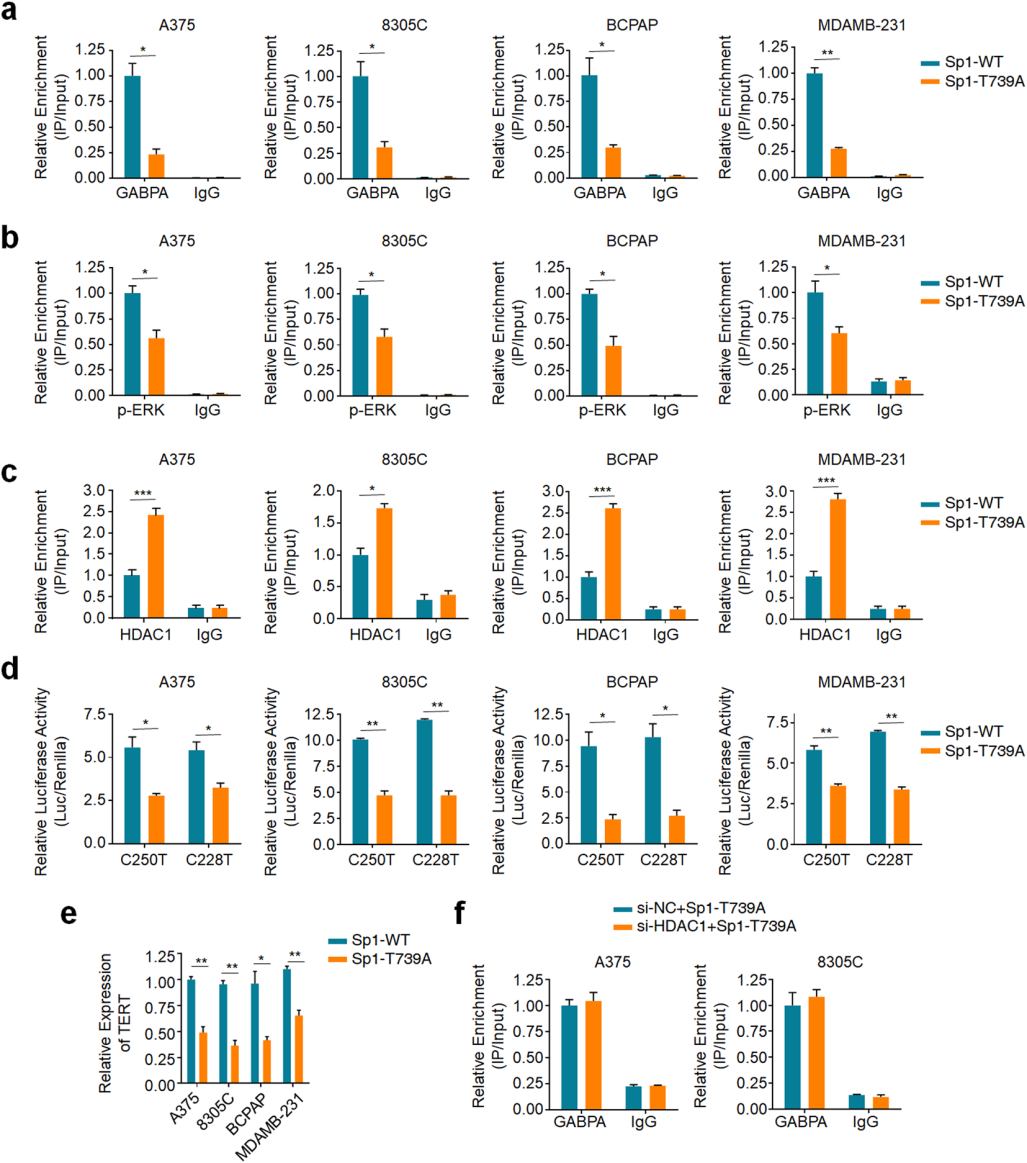
Mutation of Sp1 phosphorylation site attenuates GABPA binding and the activation of mutant *TERT* promoter. The indicated cells were transfected with wild-type (Sp1-WT) or mutant Sp1 (Sp1-T739A) expression construct, and ChIP-qPCR assay was then performed to analyze the recruitment of GABPA (**a**), p-ERK (**b**), and HDAC1 (**c**) to mutant *TERT* promoter. **d** In vitro luciferase assay was performed to determine the effect of Sp1 phosphorylation on the activity of mutant *TERT* promoter in the indicated cells. **e** qRT-PCR assay was performed to evaluate the effect of Sp1 phosphorylation on *TERT* expression in the indicated cells. *18S* rRNA was used as a reference gene. **f** ChIP-qPCR assay was performed to determine the effect of HDAC1 knockdown on the recruitment of GABPA at *TERT* promoter in A375 and 8305C cells expressing Sp1-T739A. Data were shown as mean ± SD. *P < 0.05; **P < 0.01; ***P < 0.001.

In this study, we engineered Sp1 overexpression plasmid and replicated mutant Sp1 in which the T739 residue was mutated to alanine using site-directed mutagenesis. To minimize the impact of endogenous wild-type Sp1, we first stably knocked down endogenous Sp1 in A375, 8305C, BCPAP, and MDA-MB-231 cells (Supplementary Fig. 7a), and subsequently ectopically expressed wild-type Sp1 (Sp1-WT) or T739A-mutant Sp1 (Sp1-T739A). The results showed that ectopic expression of Sp1-T739A or Sp1-WT did not affect GABPA expression and ERK phosphorylation (Supplementary Fig. 7b); however, ectopic expression of Sp1-T739A significantly decreased the recruitment of GABPA to *TERT* promoter compared to Sp1-WT (Fig. 4a). Consistent with reduced GABPA binding capacity, the recruitment of p-ERK to mutant *TERT* promoter also showed a significant decrease (Fig. 4b), while the recruitment of HDAC1 to mutant *TERT* promoter was expectedly increased in Sp1-T739A overexpressed cells compared to Sp1-WT overexpressed cells (Fig. 4c). This was further supported by our results that ectopic expression of Sp1-T739A significantly inhibited the activity of *TERT* promoter (Fig. 4d) and reduced *TERT* expression (Fig. 4e) in comparison with Sp1-WT. Finally, we knocked down HDAC1 in A375 and 8305 C cells expressing Sp1-T739A and found that HDAC1 knockdown almost did not affect the recruitment of GABPA to mutant *TERT* promoter compared to the control (Fig. 4f). The above data indicate that Sp1 phosphorylation is crucial for ERK signal-mediated GABPA binding and the activation of mutant *TERT* promoter.

### Sp1 and GABPA synergistically activate mutant *TERT* promoter

**Fig. 5:**
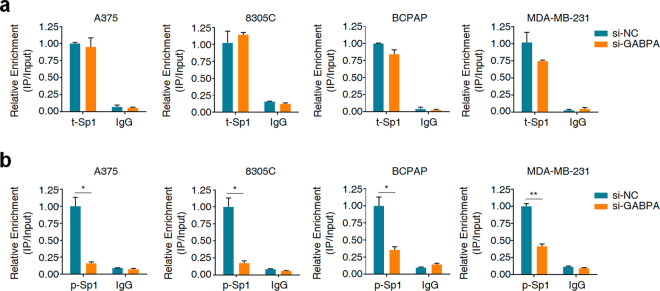
GABPA knockdown reduces the recruitment of p-Sp1 to *TERT* promoter. ChIP-qPCR assay was performed to determine the effect of GABPA knockdown on the binding of t-Sp1 (**a**) and p-Sp1 (**b**) to *TERT* promoter in the indicated cells. Data were shown as mean ± SD. *P < 0.05; **P < 0.01.

**Fig. 6:**
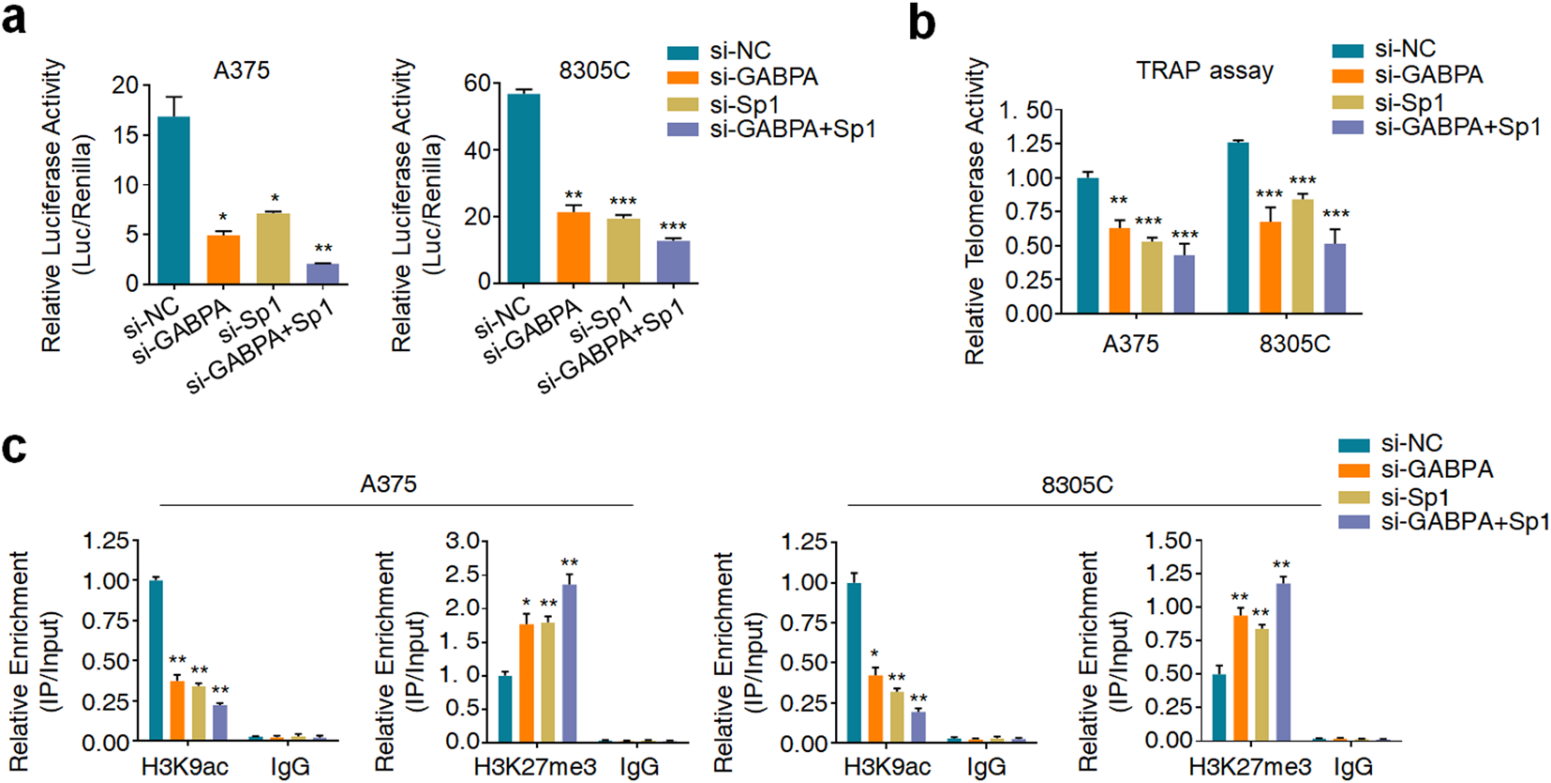
Synergistic activation of mutant *TERT* promoter by Sp1 and GABPA. **a** In vitro luciferase assay was performed to determine synergistic effect of GABPA and Sp1 on the activity of mutant *TERT* promoter in the indicated cells. **b** TARP assay was performed to determine the synergistic effect of GABPA and Sp1 on TERT reactivation. **c** ChIP-qPCR assay was performed to determine the synergistic effect of GABPA and Sp1 on histone modifications (including H3K9ac and H3K27me3) of *TERT* promoter in the indicated cells. Data were shown as mean ± SD. *P < 0.05; **P < 0.01; ***P < 0.001.

We further tested whether GABPA in turn regulates the binding of Sp1 to mutant *TERT* promoter. The results showed that knocking down GABPA in A375, 8305C, BCPAP, and MDA-MB-231 cells did not affect Sp1 expression or phosphorylation (Supplementary Fig. 8), while significantly reduced the recruitment of p-Sp1, but not t-Sp1, to *TERT* promoter (Fig. 5). Given that p-Sp1 and GABPA can be recruited to mutant *TERT* promoter with high cooperativity, thus we suppose that Sp1 and GABPA synergistically activate mutant *TERT* promoter in cancer cells carrying both *BRAF*^*V600E*^ and *TERT* promoter mutations. The results showed that knockdown of either Sp1 or GABPA significantly reduced mutant *TERT* promoter-driven luciferase activity (Fig. 6a) and telomerase activity (Fig. 6b), while dual knockdown of Sp1 and GABPA induced a further decrease relative to individual knockdown (Fig. 6a, b).

**Fig. 7:**
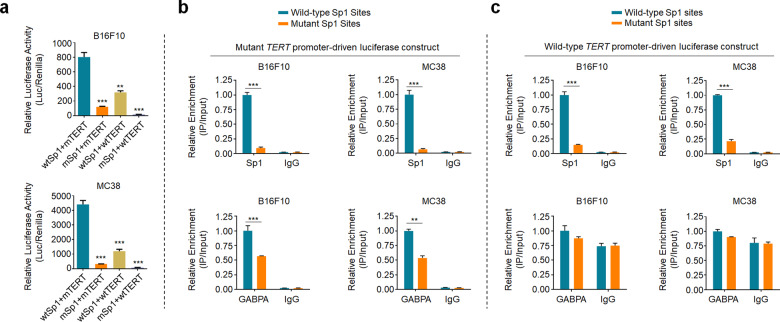
Mutations of Sp1 binding sites reduce *TERT* promoter activity and GABPA recruitment. **a** In vitro luciferase assay was performed to determine the effect of mutations of Sp1 binding sites on the activity of *TERT* promoter in the indicated cells. wtSp1 wild-type Sp1 binding sites, mSp1 mutant Sp1 binding sites, mTERT *TERT* promoter with C250T mutation, wtTERT wild-type *TERT* promoter. Mutant (**b**) and wild-type (**c**) *TERT* promoter-driven luciferase constructs with mutant or wild-type Sp1 binding sites were transfected into B16F10 and MC38 cells, and the ChIP-qPCR assays were then performed to determine the effect of the presence of Sp1 binding sites on the recruitment of Sp1/GABP to *TERT* promoter in the indicated cells. Data were shown as mean ± SD. **P < 0.01; ***P < 0.001.

Given that mutant *TERT* promoter generally exhibits active chromatin status^9^, we next determined the contribution of Sp1 or GABPA to epigenetic modifications at the *TERT* promoter. As shown in Fig. 6c, either Sp1 or GABPA knockdown caused a reduced active chromatin mark (H3K9ac) and an increased inactive chromatin mark (H3K27me3) at mutant *TERT* promoter, while dual knockdown of Sp1 and GABPA leaded to a more robust effect. Meanwhile, we established a system to determine the cooperation between GABPA and Sp1. Briefly, as shown in Supplementary Fig. 9, we found that mouse Sp1 and GABPA proteins share high homology to those in human (93.6% and 96.0%, respectively) by analyzing amino acid sequences. We next destroyed all Sp1 binding sites in mutant or wild-type promoter of human *TERT* gene, and inserted them into the luciferase constructs (Supplementary Fig. 10). Dual-luciferase assays were then performed in two mouse cancer cell lines (B16F10 and MC38). The results showed that mouse GABPA and Sp1 could bind to and activate the human *TERT* promoter (Fig. 7a). Besides, we found that mutant Sp1 binding sites led to reduced luciferase activity in both mutant and wild-type *TERT* promoter (Fig. 7a).

To determine the effect of Sp1 binding sites within the *TERT* promoter on the binding capacity of GABPA, we first validated that ChIP-qPCR primers were specific for amplifying human *TERT* promoter, but not mouse *TERT* promoter (Supplementary Fig. 11). Next, we performed ChIP-qPCR assays in B16F10 and MC38 cells transfected with different luciferase constructs. The results showed that mutations of Sp1 binding sites markedly reduced the binding of Sp1 and GABPA to mutant *TERT* promoter; however, mutations of Sp1 binding sites only reduced the recruitment of Sp1 to wild-type *TERT* promoter, while almost did not affect the binding of GABPA to wild-type *TERT* promoter (Fig. 7b, c). These data further support our conclusion that the cooperation between Sp1 and GABPA contributes to *TERT* activation. Besides, we also validated regulatory effect of HDAC1 on epigenetic modifications of *TERT* promoter (Supplementary Fig. 12), which was consistent with a previous study^20^. These data indicate that the interaction between BRAF^V600E^/ERK/Sp1/HDAC1 signaling and GABPA synergistically activates mutant *TERT* promoter, consequently enhancing *TERT* transcription.

## Discussion

It is the fact that TERT is a strong oncoprotein, which not only catalyzes the elongation of telomeres^37^, but also directly regulates the tRNA expression and enhances the proliferative capacity of cancer cells^4^. Thus, fully understanding of the mechanisms that lead to TERT reactivation will provide a potential therapeutic strategy or a biomarker for stratifying tumors. Recently, *TERT* promoter mutations have been frequently found in multiple cancer types and predict poor patient prognosis^6,38,39^. These activating mutations in *TERT* promoter generate de novo ETS binding sites, and specially recruit transcription factor GABPA, thereby leading to increased expression of TERT^8^. In addition to *TERT* promoter mutations, *BRAF*^*V600E*^ mutation also has an important role in different types of cancer, particularly melanomas and thyroid cancers^40–42^. Increasing evidences have shown that co-occurrence of *BRAF*^*V600E*^ and *TERT* promoter mutations is frequently seen in multiple cancer types, and predicts more aggressive characteristics^40,43^, indicating a strong correlation between these two frequent genetic alterations in tumorigenesis and cancer progression.

Evidently, constitute activation of BRAF signal leads to increased recruitment of p-ERK to mutant *TERT* promoter, where it provokes Sp1 phosphorylation, thereby facilitating the dissociation of HDAC1 and maintaining an active chromatin state for TERT reactivation^20^. However, it remains elusive how activated ERK selectively binds to mutant *TERT* promoter, but not wild-type one. Considering that GABPA can selectively bind to the de novo EBS created by C228T/C250T mutation^8^, thus we speculate that GABPA mediates selective recruitment of activated ERK to mutant *TERT* promoter. Our data showed that GABPA knockdown substantially reduced the recruitment of p-ERK to mutant *TERT* promoter in cancer cells carrying both *BRAF*^*V600E*^ and *TERT* promoter mutations. Further studies found that catalytic subunit GABPB rather than GABPA is more likely to directly interact with p-ERK. In addition, a previous study showed that BRAF^V600E^ could upregulate GABPB expression by ERK/Fos signaling axis, and subsequently enhanced *TERT* transcription^19^, as supported by our data that specific MEK inhibitor trametinib downregulated the expression of GABPB1, but not GABPB2. Taken together, our results indicate that GABP tetramer mediates selective binding of p-ERK to mutant *TERT* promoter.

The above findings showed that GABPA regulated the binding of p-ERK to mutant *TERT* promoter. On the other hand, activated ERK in turn can regulate the recruitment of GABPA to mutant *TERT* promoter. Our results showed that the blockade of BRAF^V600E^/ERK signaling markedly decreased the binding capacity of GABPA on mutant *TERT* promoter; however, the underlying mechanism remains elusive. Increasing evidences have demonstrated that the recruitment and transcriptional activity of ETS family members to their target sequence can be regulated by interacting with co-regulatory partners^26–30^. By sequence analysis, we found the close proximity of the binding sites of GABPA and Sp1 on mutant *TERT* promoter. Besides, a previous study has indicated that p-ERK can promote HDAC1 dissociation from Sp1/HDAC1 complex through phosphorylating Sp1, thereby activating *TERT* transcription^20^. Thus, we suppose that activated ERK enhances the recruitment of GABPA to mutant *TERT* promoter by promoting Sp1 phosphorylation and the consequent dissociation of HDAC1. Expectedly, our data showed that inhibition of BRAF^V600E^-mediated MAPK/ERK signaling reduced p-Sp1 levels and the recruitment of p-Sp1 to *TERT* promoter. Besides, Sp1 knockdown substantially reduced the recruitment of GABPA to mutant *TERT* promoter and regulatory effect of GABPA on *TERT* promoter activity, and these effects could be abolished by the blockade of ERK activation.

Considering that the binding of HDAC1 to Sp1 may potentially pose a steric hindrance for the interaction between GABPA and Sp1, thus we speculate that activated ERK may promote the interaction of Sp1 with GABPA, and the consequent recruitment of GABPA to mutant *TERT* promoter by Sp1 phosphorylation-mediated HDAC1 dissociation. This was supported by our data that inhibition of ERK activation by BRAF knockdown or trametinib treatment enhanced the interaction between Sp1 and HDAC1, and the binding of HDAC1 to *TERT* promoter, while attenuated the interaction between Sp1 and GABPA. Consistently, HDAC1 knockdown expectedly enhanced the interaction between Sp1 and GABPA. These results support the above hypothesis that p-ERK promotes the binding of GABPA to mutant *TERT* promoter by Sp1 phosphorylation. Further studies revealed that site-specific phosphorylation of Sp1 (such as T739) was required for ERK signal-mediated GABPA binding and the consequent activation of mutant *TERT* promoter.

Binding of Sp1 to *TERT* promoter is crucial for *TERT* promoter activity. It has been reported that Sp1 and Sp3 can recruit HDAC1 to *TERT* promoter in normal human somatic cells, thereby repressing *TERT* transcription^44^, while Sp1 overexpression has been demonstrated to transactivate TERT in cancer cells^45^. As supported, our data showed that both Sp1 and GABP were involved in the BRAF^V600E^/ERK-elicited epigenetic regulation on mutant *TERT* promoter, which is crucial for TERT reactivation. In addition, we also found that Sp1 and GABP could both physiologically and functionally interact with each other to activate mutant *TERT* promoter in a synergistic manner.

**Fig. 8:**
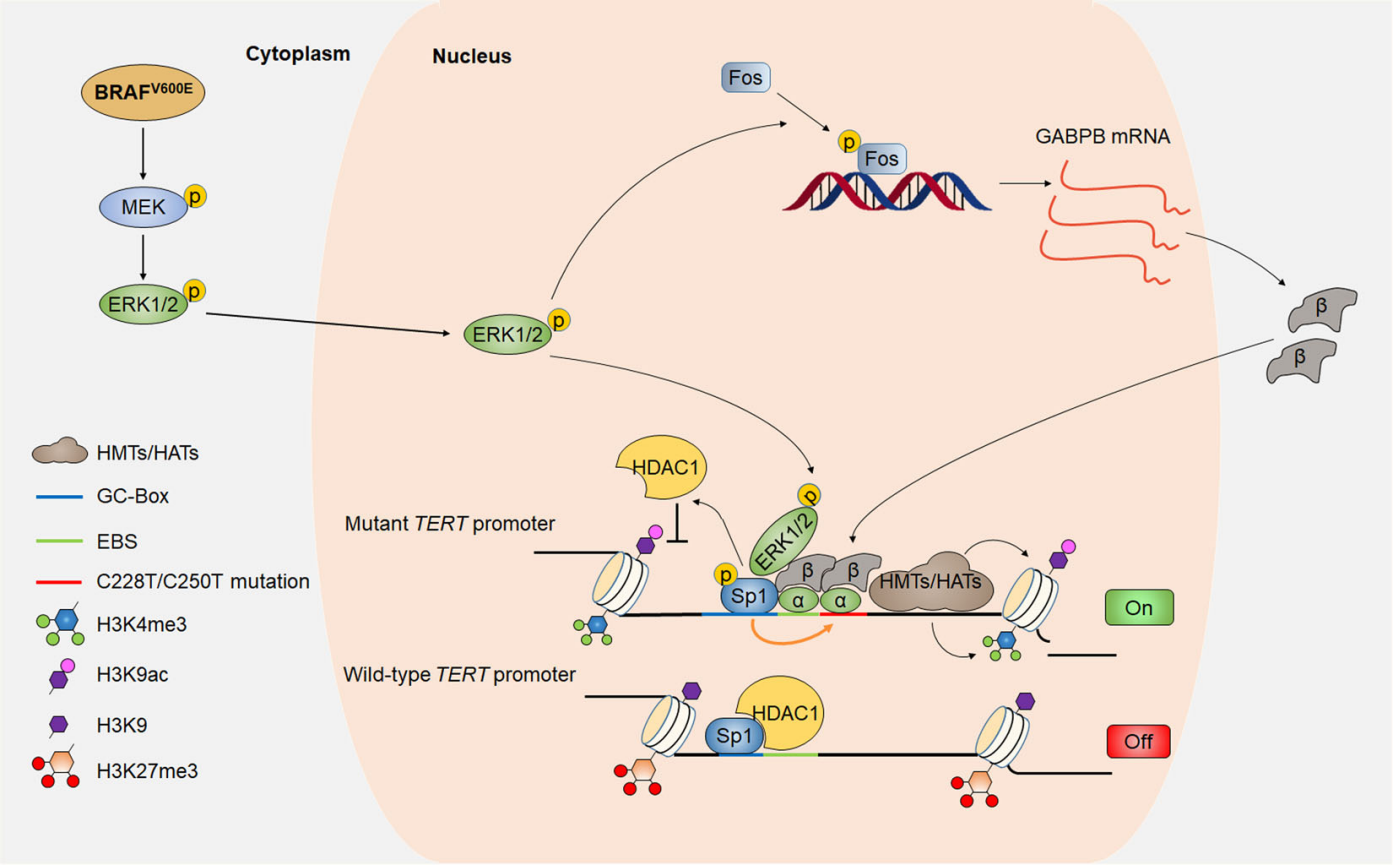
A schematic model showing the activation of the mutant *TERT* promoter by the synergistic interaction between Sp1 and GABPA. In BRAF^V600E^-driven cancer cells, GABP tetramer mediates the recruitment of activated ERK to mutant *TERT* promoter. In turn, activated ERK facilitates the interaction between Sp1 and GABPA by phosphorylating Sp1 and subsequently promoting HDAC1 dissociation from Sp1/HDAC1 complex, further enhancing the binding of GABPA to mutant *TERT* promoter. Synergistic interaction between Sp1 and GABPA will maintain an active chromatin state in mutant *TERT* promoter.

Our study supports a model (as shown in Fig. 8) in which GABP mediates the regulation of oncogenic BRAF^V600E^/ERK signaling on mutant *TERT* promoter through recruiting p-ERK. In turn, activated ERK facilitates the interaction between Sp1 and GABPA by promoting Sp1 phosphorylation and the consequent dissociation of HDAC1, thereby stabilizing the binding of GABPA to mutant *TERT* promoter. Sp1 and GABP cooperatively maintain an active chromatin state in mutant *TERT* promoter. In conclusion, the present study reveals a novel mechanism underlying synergistic effect of *BRAF*^*V600E*^ and *TERT* promoter mutations, two hotspot genetic altercations in human cancers, on TERT reactivation.

## Methods

### Reagents

MEK inhibitor trametinib (GSK1120212) was purchased from Selleck Chemicals LLC (Houston, TX, USA), and was dissolved in dimethyl sulfoxide (DMSO) with a stock concentration of 10 mM. The stock solution was diluted to 200 nM and stored at −80 °C before use.

### Cancer cell lines

Human thyroid cancer cell lines 8305C and BCPAP were kindly provided by Dr. Haixia Guan (The First Affiliated Hospital of China Medical University, Shenyang, China). Human melanoma cell line A375, gastric cell line AGS, colon cancer cell line RKO and mouse MC38 and B16F10 cells were obtained from the American Type Culture Collection (ATCC) (Manassas, VA, USA). Human breast cancer cell line MDA-MB-231 was obtained from Shanghai Cell Bank, Chinese Academy of Sciences (Shanghai, China). A375, AGS, RKO, MC38, and B16F10 cells were cultured in DMEM medium with 10% FBS (Fetal Bovine Serum). BCPAP, 8305C, and MDA-MB-231 cells were maintained in RPMI-1640 medium with 10% FBS. The *BRAF*^*V600E*^ and *TERT* promoter mutations in all cell lines have been analyzed by Sanger sequencing. All cell lines were mycoplasma free.

### siRNA/shRNA transfection

siRNAs for target genes or negative control were obtained from GenePharma (Shanghai, China) or RiboBio Co., Ltd. (Guangdong, China), and the sequences were presented in Supplementary Table 1. Cells were plated to 70% confluency and transfected using x-Tream siRNA transfection reagent (Roche) with a final concentration of 50 nM. Maximal knockdown efficiency was achieved by selecting among three different sequences. shRNA targeting Sp1 (sh-Sp1) and control shRNA (sh-NC) were purchased from GenePharma (Shanghai, China) and the sequences were also presented in Supplementary Table 1. Briefly, cells were exposed to the lentivirus for 24 h with the presence of 8 μg/mL polybrene. Positive clones were selected by adding 1 μg/mL puromycin (Sigma) for 7 days. The stable knockdown efficiency was confirmed by western blot and qRT-PCR assays. Each experiment was carried out in triplicate.

### RNA extraction and quantitative RT-PCR (qRT-PCR)

Total RNA isolation, cDNA synthesis, and qRT-PCR were performed as previously described^46^. In brief, total RNA from harvested cells were extracted with Trizol reagent (Takara) according to the manufacturer’s protocol. First-strand cDNA was prepared with 500 ng total RNA by Revert Aid First Strand cDNA Synthesis Kit (ThermoScientific). q-RTPCRs were performed on a CFX96 real-time PCR-detection System (Bio-Rad Laboratories) using KAPA SYBR FAST qPCR Master Mix (Sigma). The mRNA expression of the indicated genes was normalized to *β-actin*. The primer sequences were listed in Supplementary Table 2. Each assay was repeated in triplicate.

### Western blot analysis

The detailed procedure was carried out as previously described^47^. The harvested cells were lysed in ice-cold RIPA buffer with protease inhibitors. The protein lysates were separated on SDS–PAGE and then transferred to PVDF membranes (Roche Diagnostics). After blocked for 2 h in 5% bovine serum albumin (BSA) in 1 × TBS-T (0.5% Tween-20), the membranes were then incubated at 4 °C overnight with the indicated primary antibodies. Horseradish peroxidase (HRP)-conjugated secondary antibodies along with an ECL kit (GE Healthcare/Amersham Pharmacia Biotech, #32106) were used to detect protein signals. Multiple exposures were taken using the Western Bright ECL detection system (Advansta, CA) to select images within the dynamic range of the film (GE Healthcare Amersham Hyperfilm ECL, #28906838). Signals were normalized to GAPDH bands. The dilution ratio of antibodies for western blot analysis were shown in Supplementary Table 3 (the fourth column). All blots derive from the same experiment and were processed in parallel. Antibody information was listed in Supplementary Table 3.

### Dual-luciferase reporter assay

The C228T/C250T-mutant and wild-type TERT-pGL4.10 luciferase reporter plasmids were generated and kindly provided by Prof. Levi A. Garraway (Dana-Farber Cancer Institute, Boston, MA, USA)^48^. Luciferase activity analysis was performed according to standard procedures. Briefly, cells were plated to 70% confluency and transfected with pRL-TK and C228T-TERT-Luc or C250T-TERT-Luc plasmids using X-tremeGENE HP DNA transfection reagent (Roche) in a 12-well plate. For siRNA treatment, cells were transfected with different siRNAs for 24 h before transfection with luciferase plasmids. For trametinib treatment, cells were treated with 100 nM trametinib or DMSO for 24 h after transfection with luciferase plasmids. For overexpression experiments, cells were co-transfected with Sp1-WT or -T739A expression plasmid and luciferase plasmids. The above cells were collected 48 h post-treatment, and luminescence intensity was detected on EnSpire Multimode Plate Reader (PerkinElmer) using the dual-luciferase reporter assay system (Promega) according to the manufacturer’s instructions. The data were normalized against pRL-TK luciferase activity. Each assay was repeated in triplicate.

### Chromatin immunoprecipitation (ChIP)

The ChIP assay was performed to evaluate the recruitment of phosphorylated ERK (p-ERK), GABPA, and Sp1 to mutant or wild-type *TERT* promoter using the Pierce Magnetic ChIP Kit (Pierce Biotechnology). The detailed procedure was described as previously^49^. Briefly, the harvested cells (about 1–2 × 10^7^cells) were cross-linked using formaldehyde (final concentration 1% vol/vol) for 10 min at room temperature, followed by quenching with glycine (final concentration 0.125 M) for 5 min at room temperature. The cells were lysed with membrane extraction buffer and MNase digestion buffer for 10 min, and the whole-cell lysates were then sonicated with VCX-130PB (Sonics & Materials, Inc., Newtown, CT, USA) to fragment the chromatin to an average size of 300–500 bp. 10% of total chromatin from each lysate was used as input control, and the remaining 90% of chromatin was incubated overnight with 5 μg of indicated antibodies respectively in ChIP Buffer. Non-specific IgG was used as control. Immunoprecipitated protein DNA complex was then incubated with ChIP Grade Protein A/G Magnetic Beads for 2 h at 4 °C. Chromatin was eluted in ChIP Elution Buffer, and the proteins were removed with the addition of 200 mM NaCl and proteinase K (200 μg/mL) at 65 °C for 2 h. DNA was purified and used as templates for further analysis. Primer sequences for ChIP-qPCR analysis were listed in Supplementary Table 4. Each test was run in triplicate.

### Co-Immunoprecipitation (Co-IP)

Cells with different treatments were harvested with the RIPA buffer. Whole lysates were then incubated with 2 μg of indicated primary antibodies for 1 h at 4 °C on a rotating wheel, followed by incubation with 20 μL of Protein A/G Agarose (ThermoScientific) overnight. After four washes and boiling of the beads, bead-bound proteins in the supernatants were analyzed by western blot analysis.

### TRAP assay

The TRAP assay was performed as previously described^50^ according to the manufacturer’s guidelines. In brief, 48 h after transfection with the indicated siRNAs, the harvested 5 × 10^3^ cells were collected and lysed in Lysis Buffer containing RNase inhibitor and incubated for 30 min on ice. The cell extracts were then centrifugation at 12,000 rpm for 10 min at 4 °C. The supernatants were stored at −80 °C for further analysis. The telomerase activities were then tested by SYBR Green RQ-TRAP assay. Briefly, the samples were incubated with TRAP master mix for 20 min at room temperature and amplified in 35 PCR cycles with 30 s at 95 °C and 90 s at 60 °C. The threshold cycle values were determined by standard curves. Each test was run in triplicate.

### Site-directed mutagenesis

The wild-type human Sp1 expression plasmid was obtained from Cyagen Biosciences (Guangzhou, China). Site-directed mutagenesis of Sp1 Thr739 was performed using the QuikChange Lightning Site-Directed Mutagenesis Kit (Agilent Technologies) according to the manufacturer’s guide. The mutagenesis primer sequences were listed in Supplementary Table 5. Briefly, 10 ng of wild-type Sp1 expression plasmid was amplified with mutagenesis primers for 12 cycles, followed by digestion with restricted enzyme DpnI for 1 h at 37 °C to digest the parental DNA template. Next, 2 μL of sample reaction was transferred to stbl3 competent cells following routine procedures. Positive clones carrying the favored mutation were verified and selected by Sanger sequencing.

The potential Sp1 binding sites within the *TERT* promoter were predicted by using the transcription factor motif finder database (Jaspar), and the luciferase constructs with mutant Sp1 binding sites were obtained from TsingKe Biosciences (Xi’an, China).

### Statistical analysis

Data management and analysis were performed using the SPSS 22 and GraphPad Prism 6.0. All data in this study were presented as the mean ± SD of at least three independent replicates, and the *P-*values were generated by two-tailed Student’s *t*-tests. *P* < 0.05 was considered significant.

### Reporting summary

Further information on research design is available in the Nature Research Reporting Summary linked to this article.

## Supplementary information

supplemental material

Reporting Summary Checklist

## Data Availability

The data generated and/or analyzed during the related study are described in the figshare metadata record: 10.6084/m9.figshare.13242245^51^. Data underlying the figures are named according to the figure and subfigure they underlie (e.g., Fig. 1B 1.xlsx). These are openly available and are included together with the metadata record. They contain data concerning qRT-PCR, dual-luciferase reporter assay, ChIP, and TRAP assays described in the “Methods” section. The sequence homology analysis (underlying Supplementary Fig. 8) is openly available and is also included together with the metadata record. Data for the Co-IP assay supporting Fig. 3 are in the file WB-CoIPs.tiff and are available upon request to the corresponding author.
