# Supplementary material for: Synergistic activation of mutant TERT promoter by Sp1 and GABPA in BRAFV600E-driven human cancers
Source: NPJ Precis Oncol. 2021 Jan 22;5:3. doi: 10.1038/s41698-020-00140-5 (PMC7822828; doi:10.1038/s41698-020-00140-5)
Supplement: Supplementary file 1 — supplemental material [file 41698_2020_140_MOESM1_ESM.pdf]

Supplementary Data

Supplementary Table 1. siRNA/shRNA sequences used in this study

siRNAs/shRNAs	Sequence (5' – 3')
si-GABPA #1	GCAGGGAUCUUAAGGACAATT
si-GABPA #2	GAGCCAACUUUCAUACAUATT
si-GABPA #3	GCAGAUAGCUAUUAAUCCUTT
si-GABPB1 #1	GCAAAUGGAGCUCCCUUUATT
si-GABPB1 #2	GCUGAUGUACACACGCAAATT
si-GABPB1 #3	GCUCCAUUGUCCAAUUCUUTT
si-GABPB2 #1	GGGAAAGAGGUUGCUAGAATT
si-GABPB2 #2	CCAAGGAAGCAAUGCAGAAUTT
si-GABPB2 #3	CCCUCUGGGUAAUAUCCAATT
si-HDAC1	GGCUCCUAAAGUAACAUCATT
si-Sp1 #1	AAGCGCUUCAUGAGGAGUGTT
si-Sp1 #2	CCAACAGAUUAUCACAAAUTT
si-Sp1 #3	GCCAAUAGCUACUCAACUATT
si-NC	UUCUCCGAACGUGUCACGUTT
sh-Sp1	ACTCCTTCAGCCCTTATTA
sh-NC	CCTAAGGTTAAGTCGCCCTCG

Supplementary Table 2. qRT-PCR primers used in this study

Genes	Forward (5' – 3')	Reverse (5' – 3')
β-actin	GCACAGAGCCTCGCCTT	GTTGTCGACGACGAGCG
GABPA	TGGATACAGTGCAGCGGAGTTG	GCAGAGAAGCAGTAGCCAGAGC
GABPB1	CGGCACGAGCAGGTCAAGAT	CAGCAGTACCTCTGTGGTGGAA
GABPB2	TGGTCATCCTCCAGGAAGCAAT	GCAGCCATACTCACAGGGTCAG
Sp1	CAATGGTAATGGTGGTGGTGC	CCTGGGAGTTGTTGCTGTTCT
Sp1-3' UTR	GGATGCAAGGTAGCATGGGT	TCCCACCTTCAAAGAGGCAC

Supplementary Table 3. Antibodies used in this study

Antibody	Source	Catalogue Number	Dilution ratio (Western blot)
anti-H3K9ac	Abcam	ab10812	/
anti-H3K27me3	Cell Signaling Technology	#9733	/
anti-GABPB1/2	Santa Cruz	sc-271571	1:500
anti-GABPA	Santa Cruz	sc-28312	1:500
anti-total Sp1	Cell Signaling Technology	#8535	1:3000
anti-phosphor-Sp1 (Thr453)	Abcam	ab59257	1:3000
anti-rabbit IgG	Abcam	ab6715	/
anti-mouse IgG	Santa Cruz	sc-2025	/
anti-phospho-Erk1/2	Cell Signaling Technology	#4370	1:2000
anti-total Erk	Bioworld Technology	BS6426	1:2000
anti-HDAC1	Abcam	ab7028	1:2000
anti-TERT	Santa Cruz	sc-377511	1:1000
anti-GAPDH	Abmart	M20006	1:5000

Supplementary Table 4. ChIP-qPCR primers for the *TERT* promoter

Target	Position	Forward (5’ – 3’)	Reverse (5’ – 3’)
TERT	-209/-47	GCGCTGCCTGAAACTCGC	CGTCCTGCCCCTTCACCT
Negative control	-8504/-8384	AACTACATGGTACTGGCATAA	ATGTTCTTGGCACCTTTG

Supplementary Table 5. Mutagenesis primers for Thr739 of Sp1

	Sequence (5’ – 3’)
Forward	GGCAGTGGCACTGCCGCTCCTTCAGCCCTTATTAC
Reverse	GTAATAAGGGCTGAAGGAGCGGCAGTGCCACTGCC

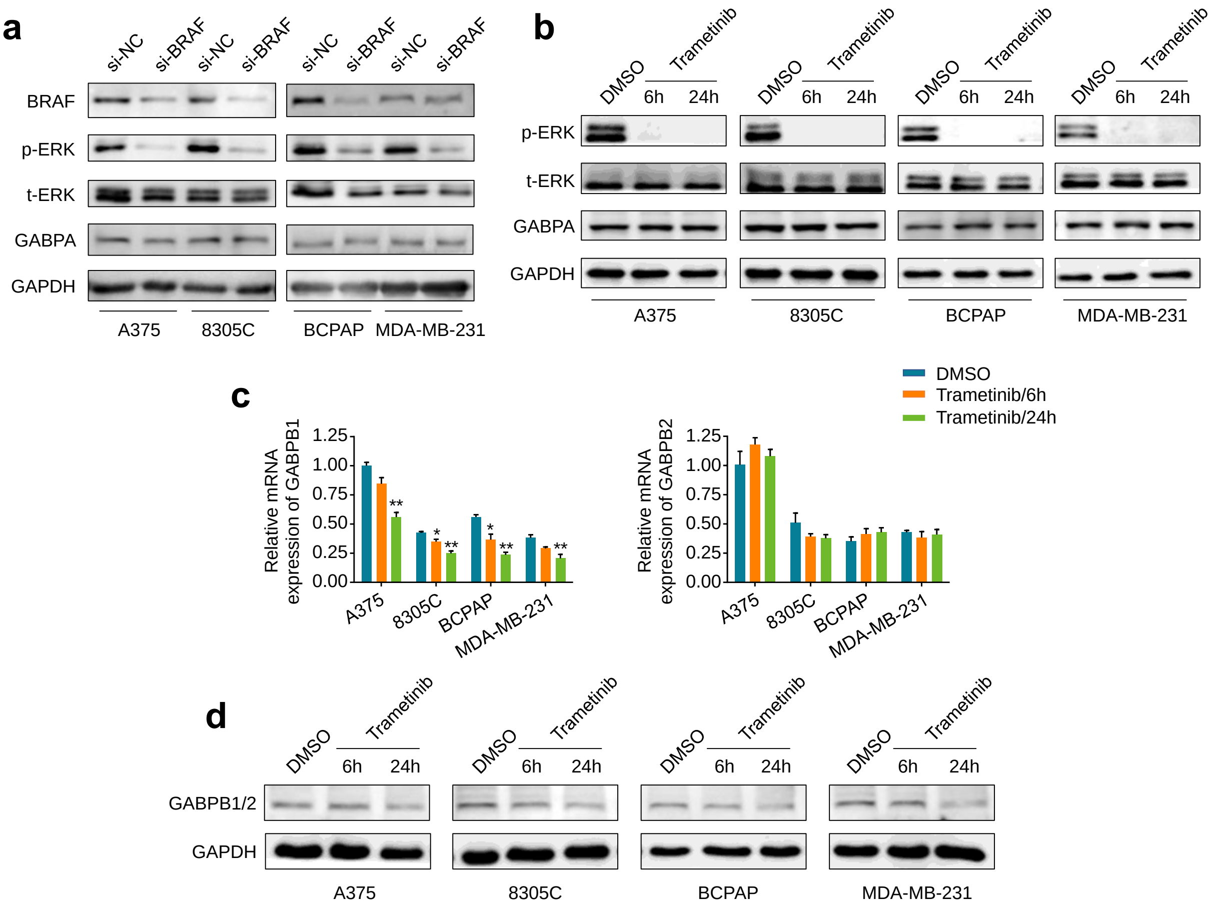

Supplementary Figure 1. The effect of the blockade of ERK signaling on the expression of GABPA or GABPB1/2. a Western blot analysis was performed to detect the expression of p-ERK, t-ERK and GABPA in A375, 8305C, BCPAP and MDA-MB-231 cells transfected with the indicated siRNAs. **b** The indicated cells were treated with DMSO or 100 nM trametinib for 6 h and 24 h, respectively, and western blot analysis was then performed to detect the expression of p-ERK, t-ERK and GABPA. The indicated cells were similarly treated with DMSO or 100 nM trametinib for 6 h and 24 h, respectively, and qRT-PCR (**c**) and western blot (**d**) assays were then performed to detect the expression of GABPB1/2. GAPDH was used as a loading control for western blot analysis. *18S* rRNA was used as a reference gene for qRT-PCR assay. Data were shown as mean ± SD. *, *P* <0.05; **, *P* <0.01.

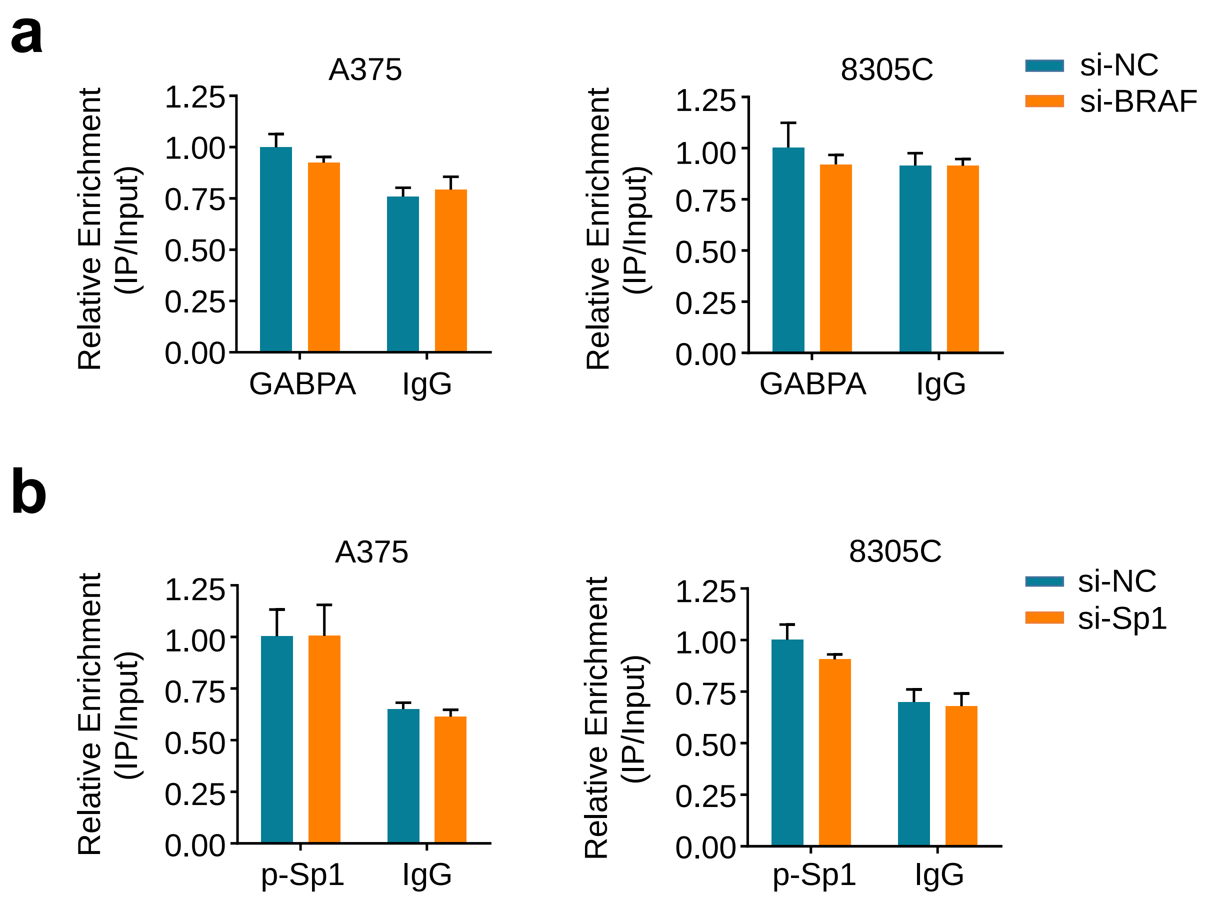

Supplementary Figure 2. The specificity of ChIP-qPCR assay was validated by determining the binding capacity of GABPA (**a**) and Sp1 (**b**) to the region lacking GABPA/Sp1 binding site. Data were shown as mean ± SD.

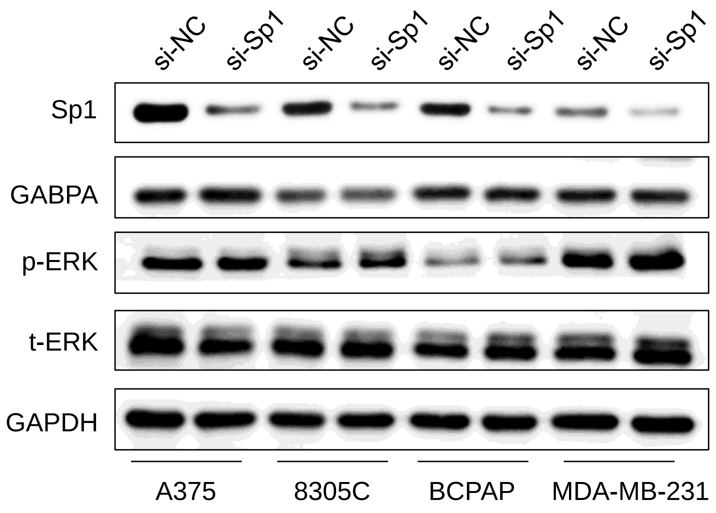

Supplementary Figure 3. Western blot analysis was performed to determine the effect of Sp1 knockdown on the levels of GABPA, p-ERK and t-ERK in the indicated cancer cells. GAPDH was used as a loading control.

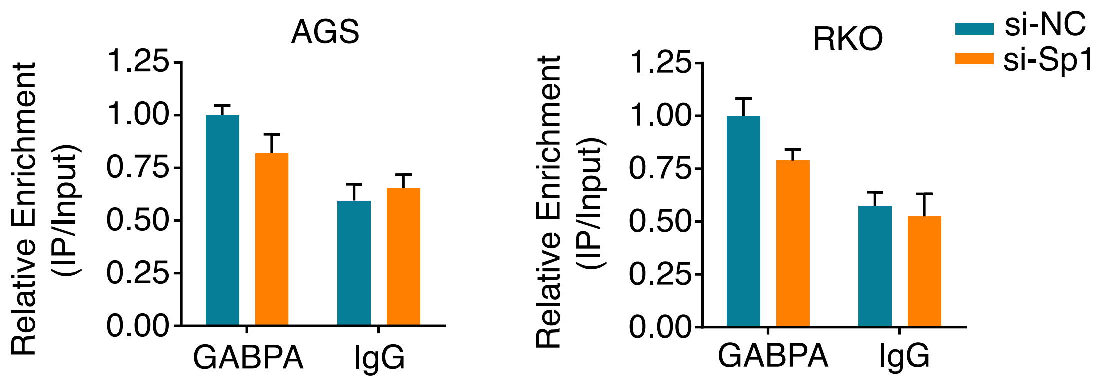

Supplementary Figure 4. ChIP-qPCR assay was performed to evaluate the effect of Sp1 knockdown on the binding of GABPA to *TERT* promoter in AGS and RKO cells. Data were shown as mean ± SD.

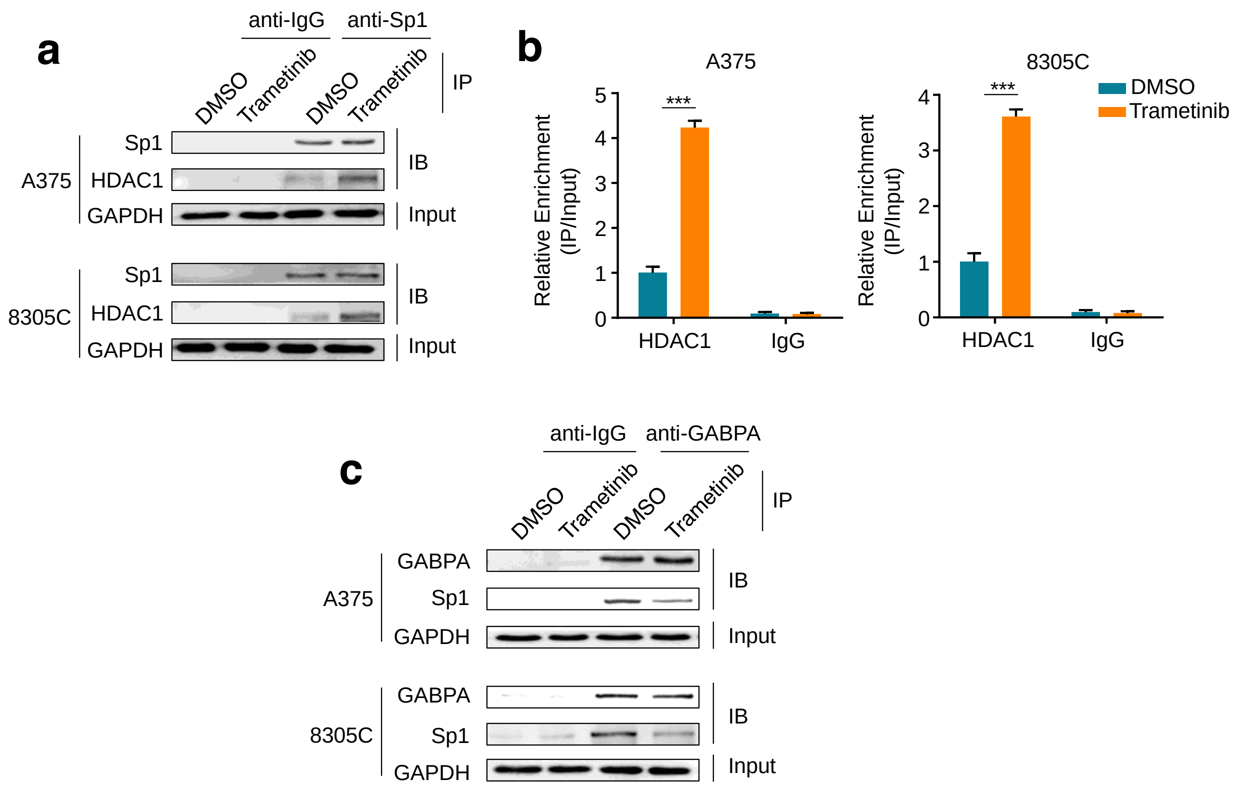

Supplementary Figure 5. a Co-IP assay of whole-cell lysates derived from the indicated cells treated with trametinib or DMSO to validate the interaction between Sp1 and HDAC1. **b** ChIP-qPCR assay was performed to evaluate the effect of trametinib treatment on the binding of HDAC1 to *TERT* promoter in the indicated cells. **c** Co-IP assay of whole-cell lysates derived from the indicated cells treated with trametinib or DMSO to validate the interaction between Sp1 and GABPA. Data were shown as mean ± SD. ***, *P* <0.001.

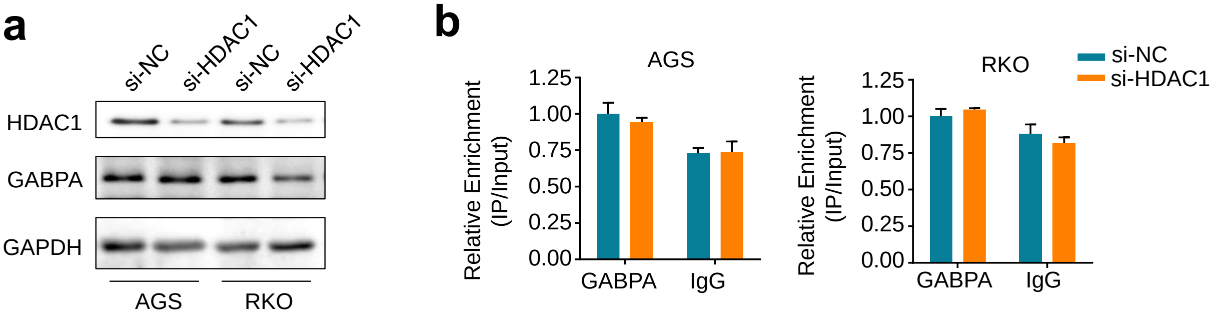

Supplementary Figure 6. a Western blot analysis was performed to determine the effect of HDAC1 knockdown on GABPA expression in AGS and RKO cells. GAPDH was used as a loading control. **b** ChIP-qPCR assay was performed to evaluate the effect of HDAC1 knockdown on the binding of GABPA to *TERT* promoter in AGS and RKO cells. Data were shown as mean ± SD.

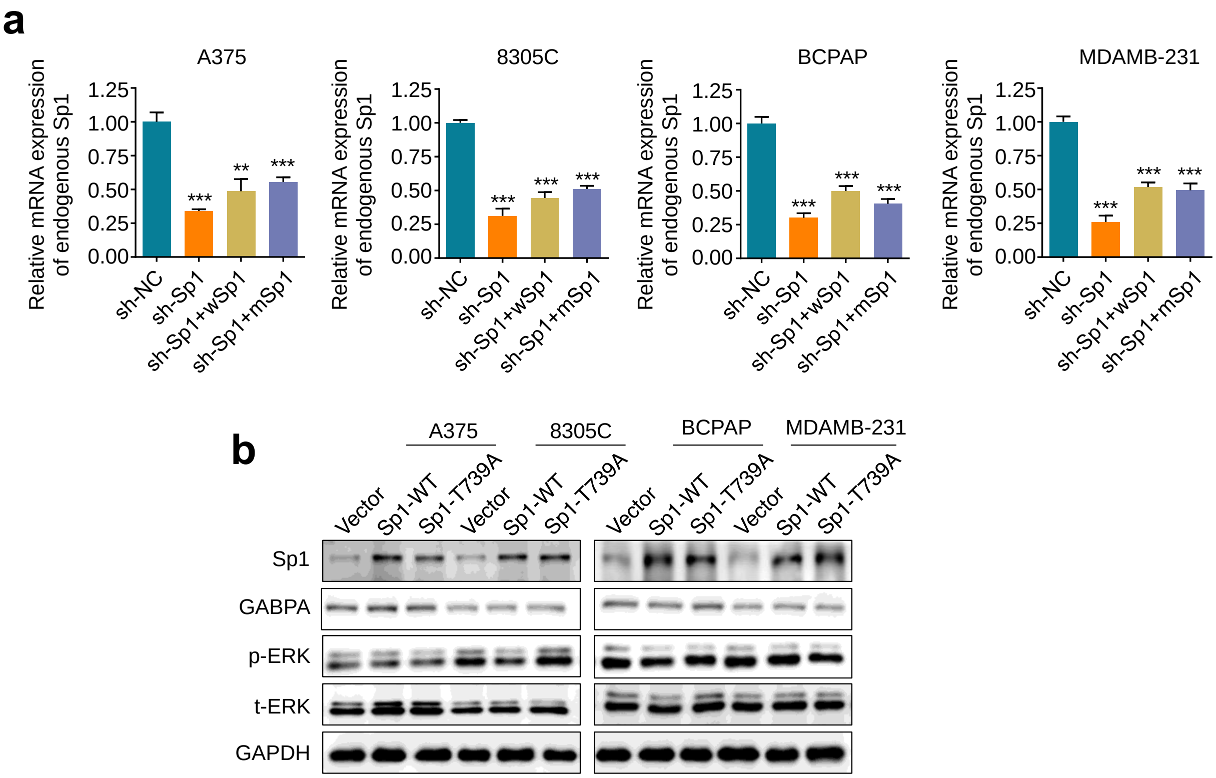

Supplementary Figure 7. The indicated cells were transfected with empty vector, wild-type (Sp1-WT) or mutant Sp1 (Sp1-T739A) expression construct. **a** qPCR assays with the primers targeting the 3'UTR of *Sp1* mRNA were performed to validate the expression of endogenous Sp1. **b** western blot analysis was then performed to detect the expression of Sp1, GABPA, p-

ERK and t-ERK in the above cells. GAPDH was used as a loading control. **, P <0.01; ***, P <0.001.

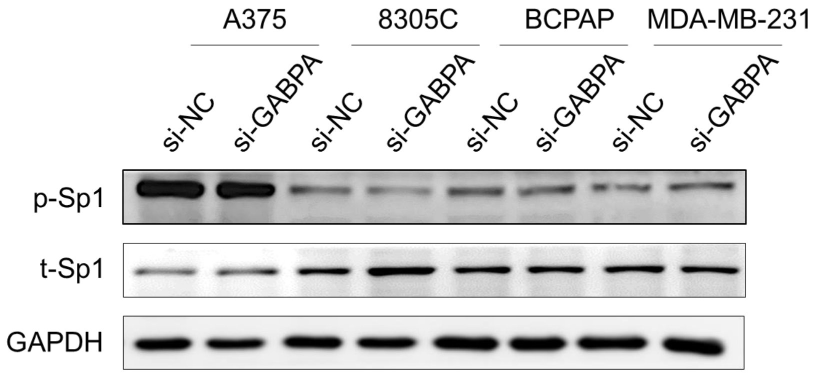

Supplementary Figure 8. Western blot analysis was performed to determine the effect of GABPA knockdown on the levels of phosphorylated Sp1 (p- Sp1) and total Sp1 (t- Sp1) in the indicated cancer cells. GAPDH was used as a loading control.

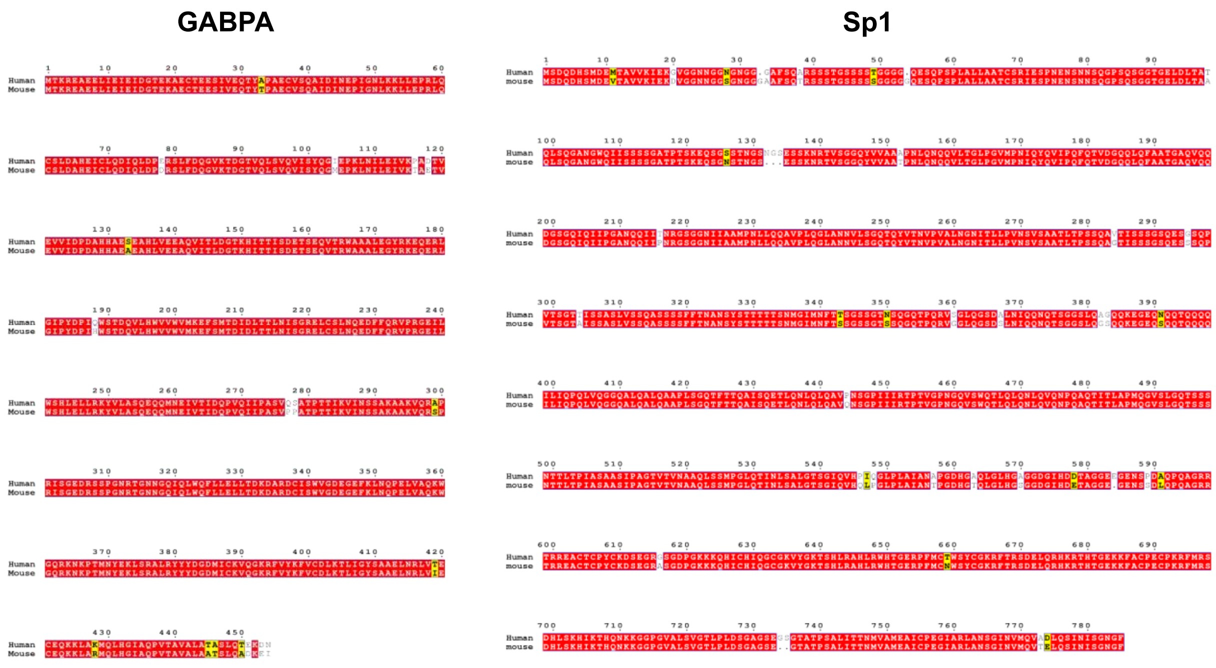

Supplementary Figure 9. Comparative analysis of amino acid sequences of GABPA and Sp1 between human and mouse.

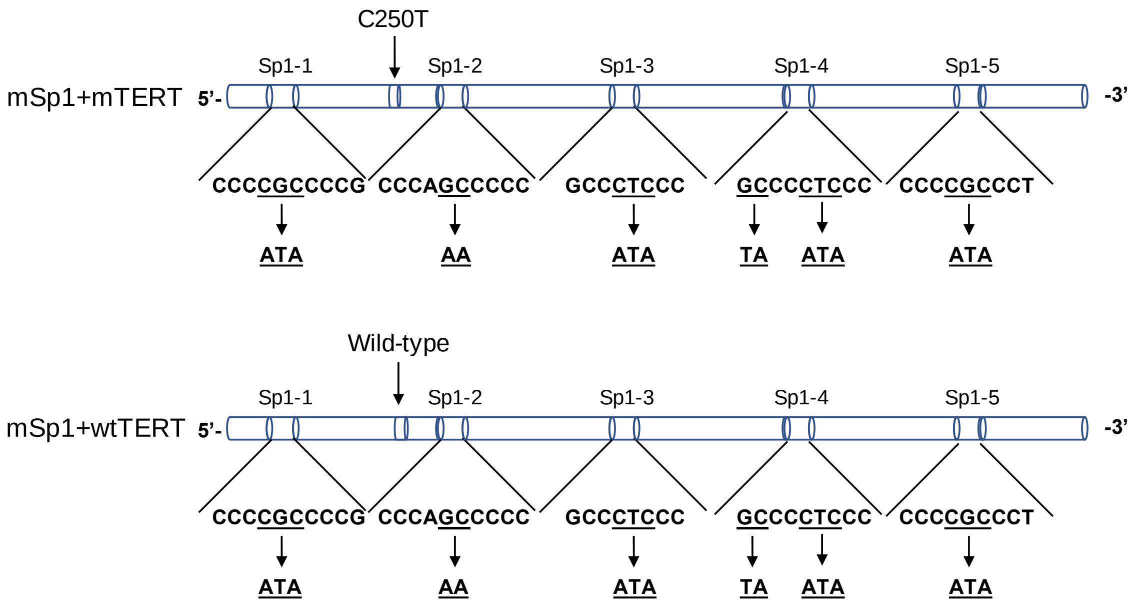

Supplementary Figure 10. A schematic model for mutations of Sp1 binding sites. mSp1, mutant Sp1 binding sites; mTERT, *TERT* promoter with C250T mutation; wtTERT, wild-type *TERT* promoter.

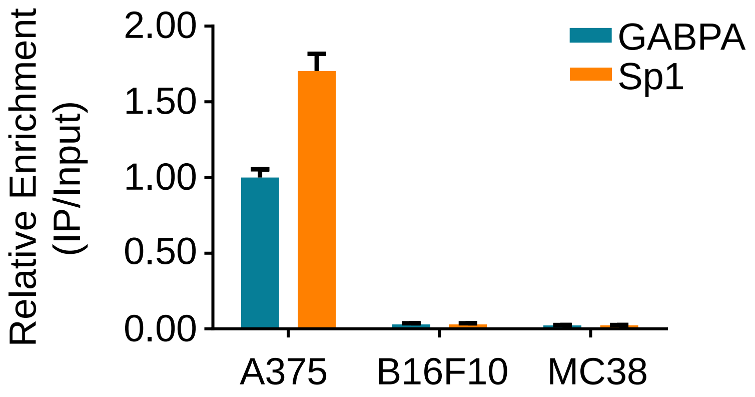

Supplementary Figure 11. Validation of the specificity of ChIP-qPCR assays for human *TERT* promoter in the indicated cells.

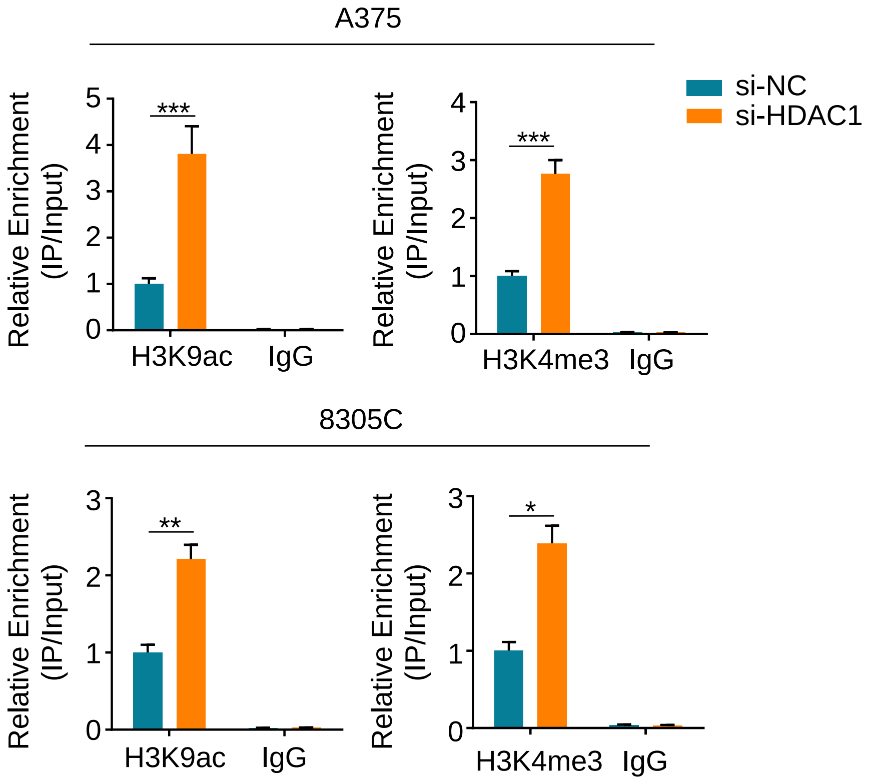

Supplementary Figure 12. ChIP-qPCR assay was performed to evaluate the effect of HDAC1 knockdown on histone modifications (including H3K9ac and H3K4me3) of *TERT* promoter in A375 and 8305C cells. Data were shown as mean ± SD. *, *P* <0.05; **, *P* <0.01; ***, *P* <0.001.

Fig1.a

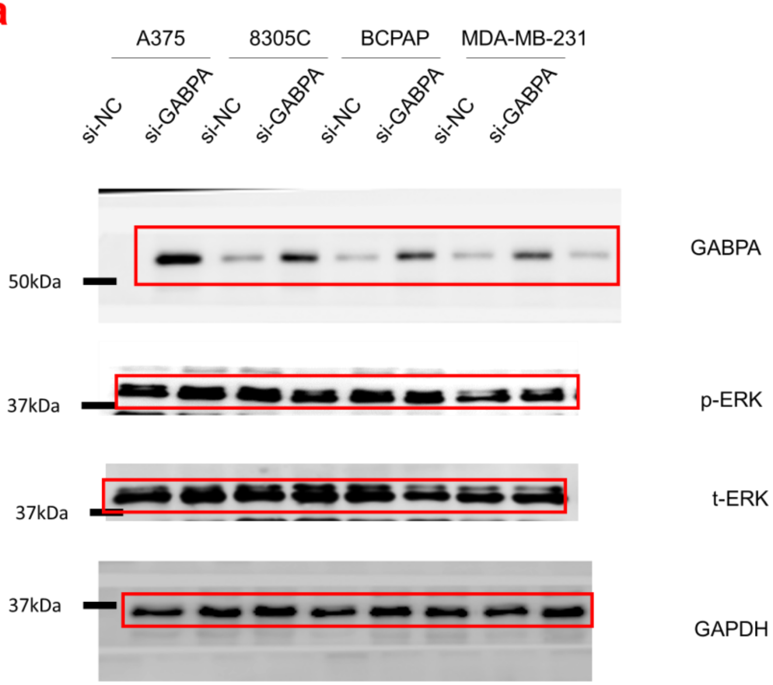

Fig.1c

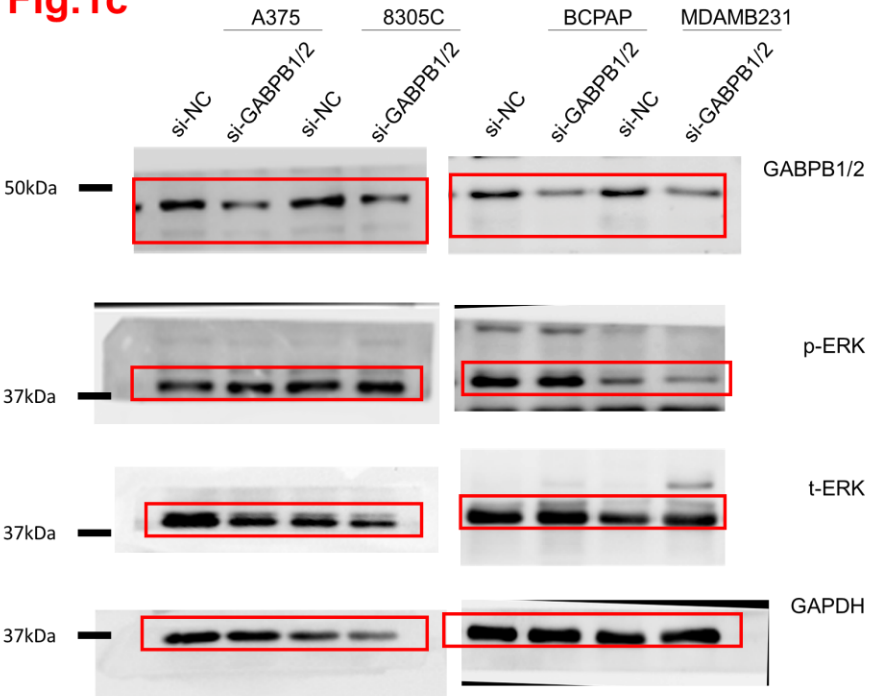

Fig.1e

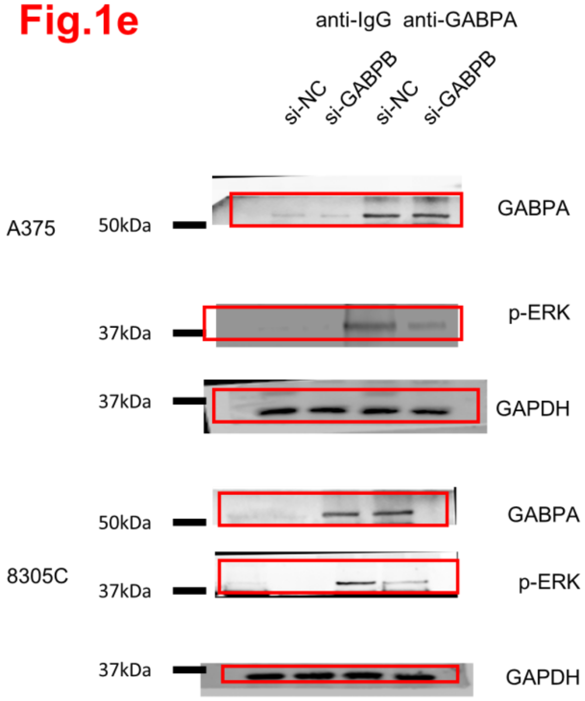

Fig.2c

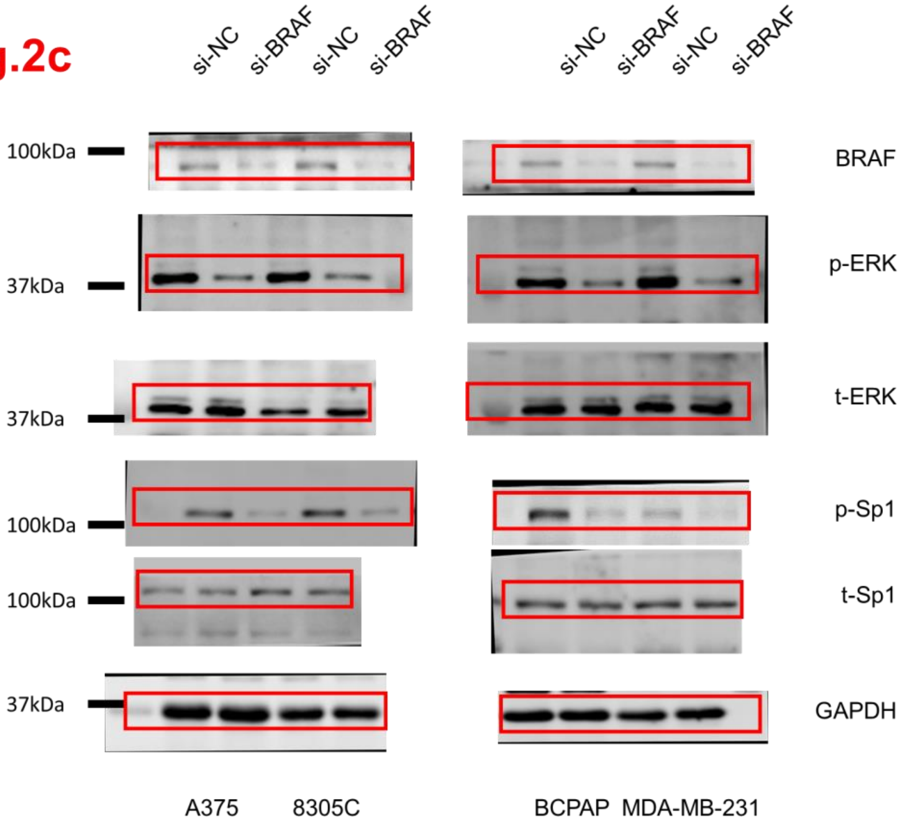
si-NC
si-BRAF
si-NC
si-BRAF
si-NC
si-BRAF
si-NC
si-BRAF
100kDa
37kDa
37kDa
100kDa
100kDa
37kDa
BRAF
p-ERK
t-ERK
p-Sp1
t-Sp1
GAPDH
A375
8305C
BCPAP
MDA-MB-231

Fig.2d

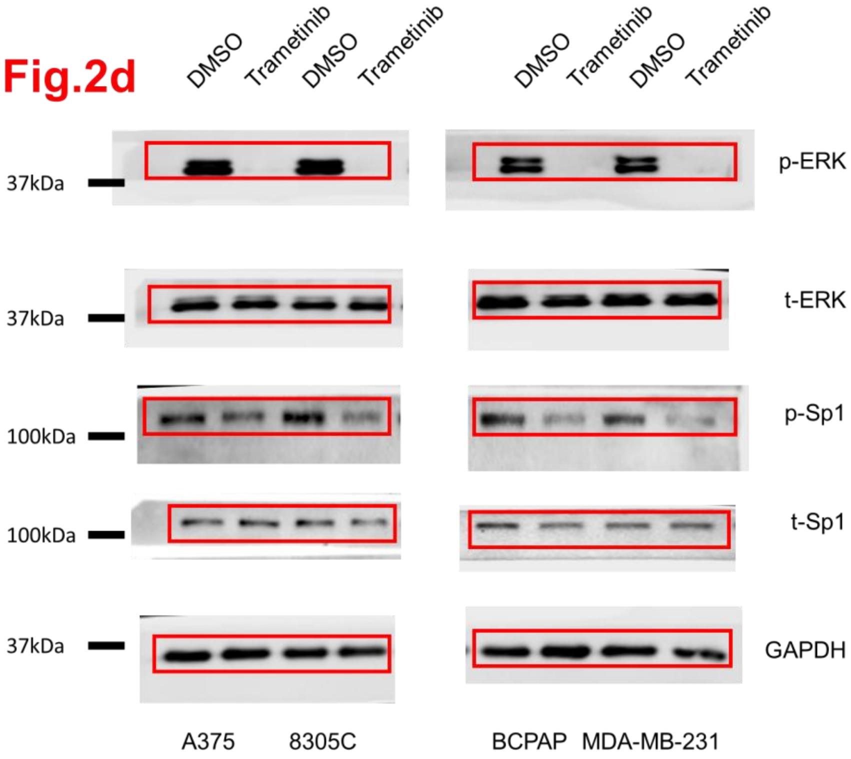
DMSO
Trametinib
DMSO
Trametinib
DMSO
Trametinib
DMSO
Trametinib
37kDa
37kDa
100kDa
100kDa
37kDa
p-ERK
t-ERK
p-Sp1
t-Sp1
GAPDH
A375
8305C
BCPAP
MDA-MB-231

Fig.3a

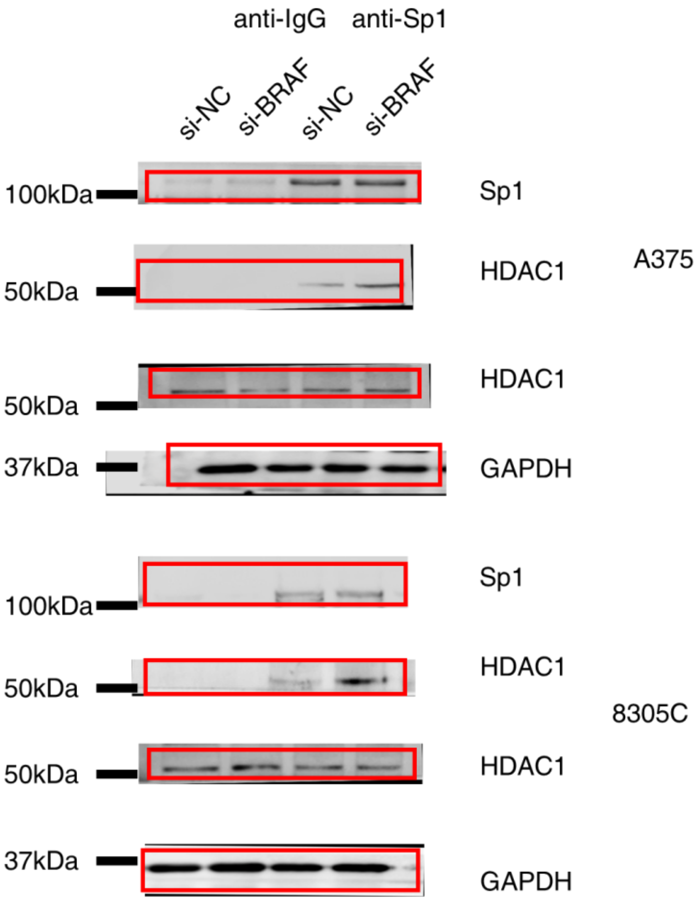

anti-IgG
anti-Sp1
si-NC
si-BRAF
si-NC
si-BRAF
100kDa
Sp1
50kDa
HDAC1
A375
50kDa
HDAC1
37kDa
GAPDH
100kDa
Sp1
50kDa
HDAC1
8305C
50kDa
HDAC1
37kDa
GAPDH

Fig.3c

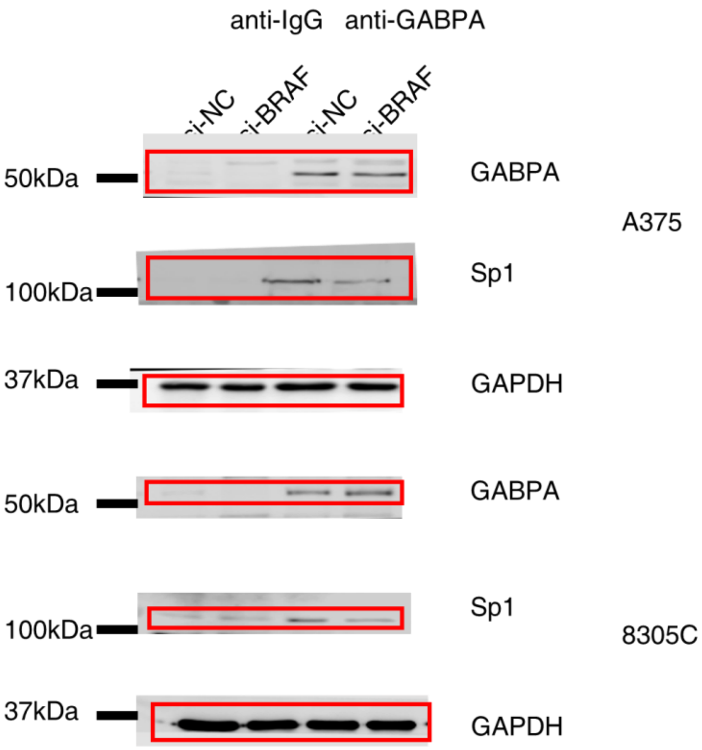

anti-IgG
anti-GABPA
50kDa
GABPA
A375
100kDa
Sp1
37kDa
GAPDH
50kDa
GABPA
100kDa
Sp1
8305C
37kDa
GAPDH

Fig.3d

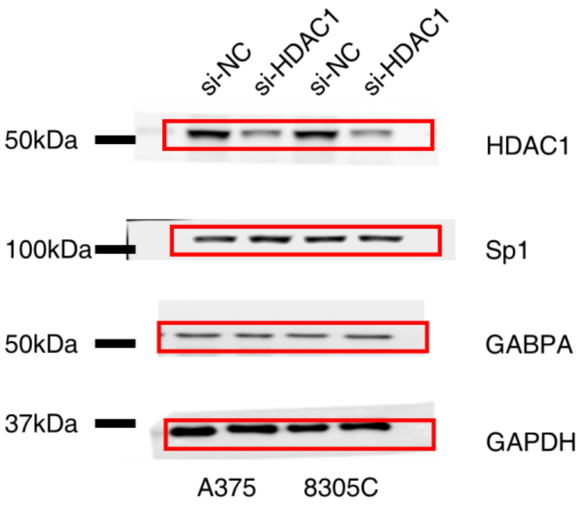
si-NC
si-HDAC1
si-NC
si-HDAC1
50kDa
HDAC1
100kDa
Sp1
50kDa
GABPA
37kDa
GAPDH
A375
8305C

Fig.3e

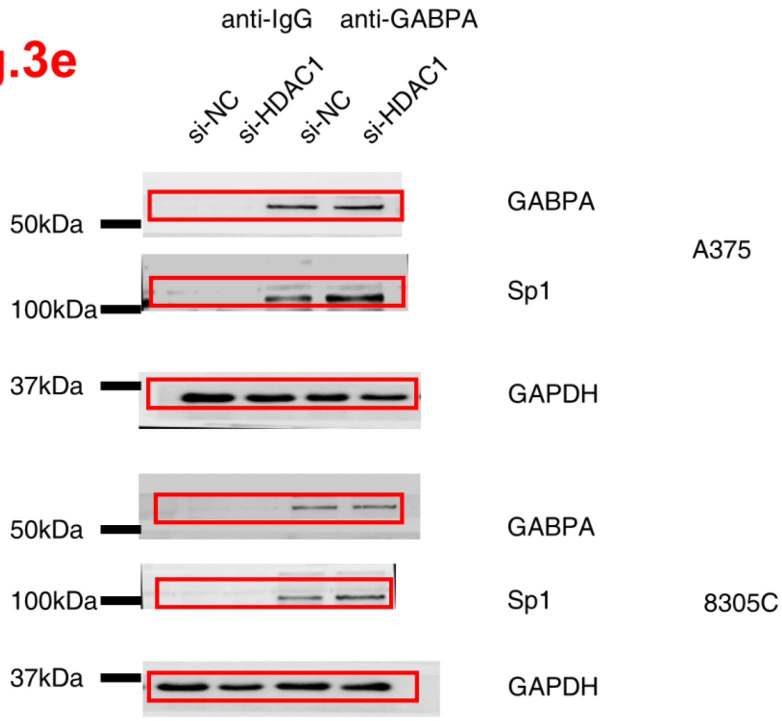
anti-IgG
anti-GABPA
si-NC
si-HDAC1
si-NC
si-HDAC1
50kDa
GABPA
A375
100kDa
Sp1
37kDa
GAPDH
50kDa
GABPA
100kDa
Sp1
8305C
37kDa
GAPDH
