# Supplementary material for: Synergistic activation of mutant TERT promoter by Sp1 and GABPA in BRAFV600E-driven human cancers
Source: NPJ Precis Oncol. 2021 Jan 22;5:3. doi: 10.1038/s41698-020-00140-5 (PMC7822828; doi:10.1038/s41698-020-00140-5)
Supplement: Supplementary file 2 — Reporting Summary Checklist [file 41698_2020_140_MOESM2_ESM.pdf]

nature research

Corresponding author(s): Peng Hou

Last updated by author(s): Oct 29, 2020

Reporting Summary

Nature Research wishes to improve the reproducibility of the work that we publish. This form provides structure for consistency and transparency in reporting. For further information on Nature Research policies, see our Editorial Policies and the Editorial Policy Checklist.

Statistics

For all statistical analyses, confirm that the following items are present in the figure legend, table legend, main text, or Methods section.

n/a	Confirmed	
☐	☒	The exact sample size (n) for each experimental group/condition, given as a discrete number and unit of measurement
☐	☒	A statement on whether measurements were taken from distinct samples or whether the same sample was measured repeatedly
☐	☒	The statistical test(s) used AND whether they are one- or two-sided<br>*Only common tests should be described solely by name; describe more complex techniques in the Methods section.*
☒	☐	A description of all covariates tested
☒	☐	A description of any assumptions or corrections, such as tests of normality and adjustment for multiple comparisons
☒	☐	A full description of the statistical parameters including central tendency (e.g. means) or other basic estimates (e.g. regression coefficient) AND variation (e.g. standard deviation) or associated estimates of uncertainty (e.g. confidence intervals)
☒	☐	For null hypothesis testing, the test statistic (e.g. F, t, r) with confidence intervals, effect sizes, degrees of freedom and P value noted<br>*Give P values as exact values whenever suitable.*
☒	☐	For Bayesian analysis, information on the choice of priors and Markov chain Monte Carlo settings
☒	☐	For hierarchical and complex designs, identification of the appropriate level for tests and full reporting of outcomes
☒	☐	Estimates of effect sizes (e.g. Cohen's d, Pearson's r), indicating how they were calculated

Our web collection on statistics for biologists contains articles on many of the points above.

Software and code

Policy information about availability of computer code

Data collection	No software was used.
Data analysis	SPSS 22 and GraphPad Prism 6.0 were used in this study.

For manuscripts utilizing custom algorithms or software that are central to the research but not yet described in published literature, software must be made available to editors and reviewers. We strongly encourage code deposition in a community repository (e.g. GitHub). See the Nature Research guidelines for submitting code & software for further information.

Data

Policy information about availability of data

All manuscripts must include a data availability statement. This statement should provide the following information, where applicable:

- Accession codes, unique identifiers, or web links for publicly available datasets
- A list of figures that have associated raw data
- A description of any restrictions on data availability

The data that support the findings of this study are available from the corresponding author upon reasonable request.

Field-specific reporting

Please select the one below that is the best fit for your research. If you are not sure, read the appropriate sections before making your selection.

☒ Life sciences ☐ Behavioural & social sciences ☐ Ecological, evolutionary & environmental sciences

For a reference copy of the document with all sections, see nature.com/documents/nr-reporting-summary-flat.pdf

Life sciences study design

All studies must disclose on these points even when the disclosure is negative.

Sample size	Disclosure is not applicable.
Data exclusions	No data were excluded.
Replication	Each assay was repeated in triplicate, and the replication were successful.
Randomization	Disclosure is not applicable.
Blinding	Investigators are blind during analysis.

Reporting for specific materials, systems and methods

We require information from authors about some types of materials, experimental systems and methods used in many studies. Here, indicate whether each material, system or method listed is relevant to your study. If you are not sure if a list item applies to your research, read the appropriate section before selecting a response.

Materials & experimental systems

n/a	Involved in the study	
☐	☒	Antibodies
☐	☒	Eukaryotic cell lines
☒	☐	Palaeontology and archaeology
☒	☐	Animals and other organisms
☒	☐	Human research participants
☒	☐	Clinical data
☒	☐	Dual use research of concern

Methods

n/a	Involved in the study	
☒	☐	ChIP-seq
☒	☐	Flow cytometry
☒	☐	MRI-based neuroimaging

Antibodies

Antibodies used	All the antibodies used in this study are listed in supplementary Table 3.
Validation	All the antibodies are validated before use.

Eukaryotic cell lines

Policy information about cell lines

Cell line source(s)	Human thyroid cancer cell lines 8305C and BCPAP were kindly provided by Dr. Haixia Guan (The First Affiliated Hospital of China Medical University, Shenyang, China). Human melanoma cell line A375, gastric cell line AGS, colon cancer cell line RKO and mouse MC38 and B16F10 cells were obtained from the American Type Culture Collection (ATCC) (Manassas, VA, USA). Human breast cancer cell line MDA-MB-231 was obtained from Shanghai Cell Bank, Chinese Academy of Sciences (Shanghai, China).
Authentication	All cell lines were used authorized.
Mycoplasma contamination	All the cell lines were tested negative for mycoplasma.
Commonly misidentified lines (See ICLAC register)	No such cell lines were used.
